# A Systematic Review of Qualitative Studies Exploring Lived Experiences, Perceived Impact, and Coping Strategies of Children and Young People Whose Parents Use Substances

**DOI:** 10.1177/15248380221134297

**Published:** 2022-11-17

**Authors:** Cassey Muir, Emma A. Adams, Vivienne Evans, Emma Geijer-Simpson, Eileen Kaner, Sophie M. Phillips, Domna Salonen, Deborah Smart, Lizzy Winstone, Ruth McGovern

**Affiliations:** 1 Newcastle University, Newcastle upon Tyne, UK; 2 Adfam, London, UK; 3 Durham University, UK; 4 Cumbria, Northumberland, Tyne and Wear NHS Foundation Trust, UK; 5 University of Bristol, UK

**Keywords:** alcohol and drugs, child abuse, anything related to child abuse, children exposed to domestic violence, domestic violence, family issues and mediators

## Abstract

Parental substance use is highly prevalent worldwide, presenting major child safeguarding and public health concerns. Qualitative research enables in-depth understanding of how young people experience parental substance use and helps inform practice and policy through illustrative cases of experiences. This review aimed to synthesize published qualitative evidence exploring the lived experiences, perceived impact, and coping strategies of children and young people whose parents use substances. International literature databases including Medline, PsycINFO, Cumulative Index to Nursing and Allied Health Literature, International Bibliography of the Social Sciences, Social Science Database, Sociology Collection, and Scopus were searched from inception to 2022, alongside grey literature searching and relevant websites. Qualitative accounts were included, provided by participants aged below 25 years. No language, date, or geographical limits were applied. A thematic synthesis of 35 studies, across 49 papers, covering over 700 children and young people’s voices, identified five overarching themes. These themes included, (a) living with the unpredictable: insecurity within the family; (b) social and emotional impact of parental substance use; (c) controlling the uncontrollable: creating safety within the family; (d) coping with and resisting the emotional and social impacts; and (e) formal and informal support. The findings emphasize that children and young people who experience parental substance use are trying to manage and mitigate vulnerabilities and be resilient to unpredictable, adverse, and often stigmatizing experiences, usually without formal support in place. Further research is needed to coproduce child-centered interventions that promote children and young people’s social and emotional resilience.

## Background

Parental substance use is highly prevalent worldwide, presenting major child safeguarding, health, and social concerns (Canfield et al., 2017). Estimates suggest that between 2 and 37% of children live with at least one parent who uses substances (European Monitoring Centre for Drugs and Drug Addiction, 2008; Galligan & Comiskey, 2019). In the United Kingdom, recent estimates suggested that around 4% or 478,000 children lived with a parent who uses alcohol or drugs in 2019 to 2020 (Children’s Commissioner’s Office, 2020). These children have been found to have poor school attendance and concentration (Díaz et al., 2008), low academic performance (Hogan & Higgins, 2001), antisocial problems (Molina et al., 2010), anxiety and depression (Gorin, 2004), as well as their own substance using and offending behaviors (Velleman & Templeton, 2016). Such impacts have also been found among young adult children who experience parental substance use (Pisinger & Tolstrup, 2022). There is also emerging evidence that this is true of parental substance use below the diagnostic threshold (Institute of Alcohol Studies, Adfam, & Alcohol Focus Scotland, 2017; McGovern et al., 2018). These children can go on to experience multiple disadvantages into adulthood, driven and exacerbated by structural risk factors such as poverty (Marmot et al., 2020).

Most existing research is cross sectional and states that young people are either vulnerable or resilient to the impacts of parental substance use, depending on several risk and protective factors (Velleman & Templeton, 2007). Risk factors can exacerbate the effect of parental substance use on young people, while protective factors can help reduce such negative impacts. Protective factors and risk factors may be individual (e.g., having high esteem or low esteem), parental (e.g., positive, and consistent parenting or negative and inconsistent parenting), familial (e.g., no other comorbid psychopathology in parents or additional comorbidities), as well as social (e.g., positive social support or no social support) (Park & Schepp, 2015; Wlodarczyk et al., 2017). Such research has been crucial in understanding factors that can promote resilience. Additionally, some young people who experience parental substance use may have less adaptive coping styles than their peers (Hussong & Chassin, 2004), while others have been found to present resilient coping strategies (Werner & Johnson, 2004). Qualitative research eliciting children and young people’s experiences of parental substance use has the potential to give a deeper, child-centered understanding of what it is like for children and young people to live with parental substance use, how it impacts them, and how they cope with their experiences. This understanding can help inform practice and policy, as well as child-focused intervention development. A small number of non-systematic reviews have examined children’s experiences of parental substance use (Adamson & Templeton, 2012; Kroll, 2004). These reviews were both limited by date and geographical restrictions or only considered parental alcohol use (Adamson & Templeton, 2012). To date, no thorough qualitative systematic review of children and young people’s experiences of parental substance use has been published.

### Aims and Objectives

This qualitative systematic review aimed to produce a child- and young person-focused account of experiences of parental substance use, perceived impact, and coping strategies. The objectives were to identify, appraise, and synthesize qualitative literature across these three areas.

## Method

The review protocol was registered with PROSPERO (Muir et al., 2019) (CRD42019137486). The international literature was searched from inception to February 2022 using electronic databases, Medline (OVID), PsycINFO (OVID), Cumulative Index to Nursing and Allied Health Literature (EBSCOhost), International Bibliography of the Social Sciences (ProQuest), Social Science Database (ProQuest), Sociology Collection (ProQuest), including, Applied Social Sciences Index and Abstracts, Sociology Database, and Sociological Abstracts, and Scopus. Key words were developed relating to the concepts, “children and young people” and “parental substance use.” Key words were mapped to relevant MeSH/thesaurus terms and truncated, exploded, or focused as appropriate, with variant spellings used (see Supplemental Materials for search strategy). Due to the difficulty of identifying relevant qualitative research (Shaw et al., 2004), a validated search filter designed to identify qualitative research was applied (DeJean et al., 2016). No language, date, or geographical limits were applied. Searches were supplemented with Google, Google Scholar, and Open Grey relevant websites and hand-searching reference lists and citations of included studies.

### Eligibility Criteria

Two reviewers independently screened all titles and abstracts using Rayyan, with specified inclusion and exclusion criteria, retrieving full papers for all potentially eligible studies, and evaluating in full text. Discrepancies at each stage were resolved by discussion or by consulting a third researcher if consensus could not be reached. Non-English papers were translated by individual’s bilingual in the language and English. Relevant data were extracted independently by two reviewers, including study design and methodology, sample characteristics, nature of parental substance use, and findings relevant to the review. Authors were contacted when articles were irretrievable online, or data were missing.

Studies were included that focused on the lived experiences, perceived impact and/or coping strategies of children and young people aged below 25 years (or where the mean age was less than or equal to 25 years) whose parent(s) used substances. Three studies (reported across eight papers) reported analysis of data from young people with an age range spanning beyond age 25 years (Backett-Milburn et al., 2008; Bancroft et al., 2004; Park & Schepp, 2017, 2018; Park et al., 2016; Wangensteen et al., 2019, [Bibr bibr95-15248380221134297][Bibr bibr96-15248380221134297]). These studies were included, but accounts from those aged under 25 were prioritized. Parental substance use included any use that had the potential to cause harm to a child or young person, with a focus upon high-risk patterns of substance use. This ranged from frequent or heavy alcohol use to any use of illicit drugs, including the misuse of legally prescribed drugs. Studies were excluded if they: mainly reported findings from looked-after children or those in custodial criminal justice settings; reported the views of others (e.g., parents or professionals) rather than of children and young people themselves; or on parental tobacco and/or caffeine use.

### Quality Assessment

Included papers were quality assessed using a two-stage process adapted from Britten and Pope (2012). Firstly, quality was assessed using the 10-item Critical Appraisal Skills Programme (CASP) Qualitative Studies Checklist, to evaluate the studies on clarity, appropriateness, rigor, and overall value (Critical Appraisal Skills Programme, 2018). See Supplemental Materials for CASP appraisal. Studies were not excluded based on quality, but a modified rating scale based on Dixon-Woods et al. (2007) and Malpass et al. (2009) was used to aid the synthesis process and decide the relevance of studies to the review. Studies were rated as (A) a key paper that was most relevant and conceptually rich, with no or few quality issues; (B) a secondary key paper, that was relevant but with limited themes and data, and/or some quality issues; or (C) satisfactory, that was less relevant to the review and/or the CASP appraisal highlighted major limitations related to the quality of reporting. Data extraction and appraisal were completed simultaneously by the lead author and checked by a second author. Any discrepancies in decisions were resolved through discussion.

### Data Synthesis

The synthesis process was led by the lead author with discussion among the research team and a public advisor. Synthesis was based on Thomas and Harden’s (2008) three-stage thematic method that moves iteratively between coding, identification of descriptive themes, and generation of analytic themes. The first stage involved familiarization of findings of each study during full-text screening and immersion through repeated reading. During data extraction and quality appraisal, the lead author listed initial ideas and then inductively generated line-by-line codes from the study findings and author interpretations using NVivo 12 management software (QSR International Pty Ltd, 2018). Next, recurring codes explaining findings across the studies, were then developed into three descriptive themes based on the main research questions: (a) lived experiences, (b) impacts, and (c) coping strategies used to manage adverse impacts. The third stage of synthesis involved identifying and mapping links between the descriptive themes to generate analytical themes that, together, made sense of children and young people’s experiences of parental substance use. Throughout the synthesis process, themes were discussed and refined among practice and policy practitioners, as well as with four young people, aged 11 to 17 years, who had experienced parental substance use, and their two support workers.

## Findings

### Description of Studies

**Figure 1.**
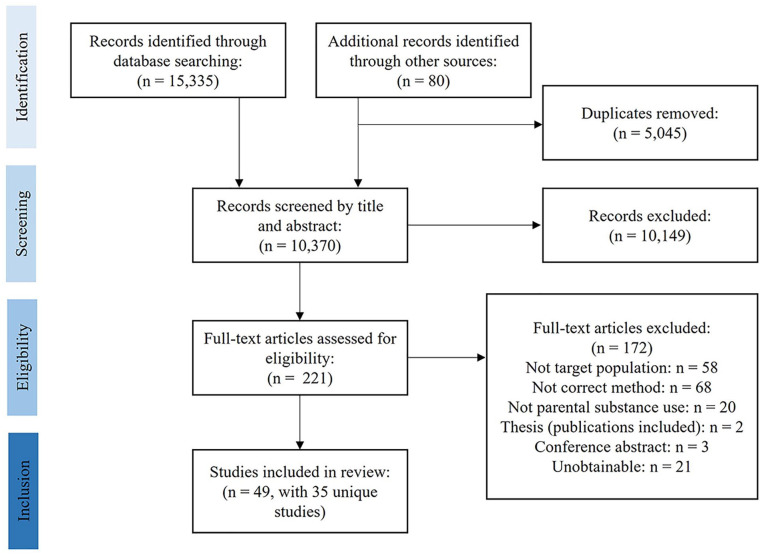
Flowchart of included studies.

Thirty-five individual studies, reported across 49 papers, were included (see Figure 1 for flowchart). Based on quality and relevance, 23 studies were rated as key papers (A and B), and 12 studies were rated as satisfactory. The synthesis of findings involved over 737 children and young people (aged 4–30 years) whose parents use(d) substances. Table 1 provides further descriptive summary characteristics of the included studies. Where reported, there were 417 female and 250 male participants. Two studies (four papers) explored Black African and American young people’s experiences (Johnson, 2013; Lewis et al., 2021; Offiong et al., 2020; Powell et al., 2021), while Ahuja et al. (2003) explored Sikh daughters’ perspectives. Ten studies (11 papers) reported that all young people were living with the parent who uses substances at the time of data collection (Ahuja et al., 2003; D’Costa & Lavalekar, 2021; Dundas, 2000; M. Hill et al., 1996; Johnson, 2013; Mudau, 2018; Ramírez Dávila et al., 2014; Reupert et al., 2012; Templeton et al., 2009; Tinnfält et al., 2018; Velleman et al., 2008). All other studies reported varied living arrangements for young people. Studies recruited samples from across 20 countries, with the majority from Europe (*n* = 21), then North America (*n* = 5), Asia (*n* = 5), Oceania (*n* = 2), South America (*n* = 1), and Africa (*n* = 1).

**Table 1.** Brief Descriptive Summaries of the 35 Included Studies With Quality Appraisal (Key Paper: A/B; Satisfactory Paper: C).

First Author (Year) and Country	Sample Size (Female), Ages	Parental Substance Use	Data Collection, Recruitment, and Analysis	Quality Appraisal
Ahuja (2003) England	*N* = 7 (7F), 17–23	Father’s alcohol use	Semi-structured interviews; specialist addiction treatment service for parents; grounded theory	C
Alexanderson (2016) Sweden	*N* = 23, 6–19	Parental substance use	Semi-structured interviews; social services/support groups for children; grounded theory	C
Bancroft (2004) Scotland (Backett-Milburn, 2008; Wilson, 2008, 2012)	*N* = 38 (20F), 15–27	Parental substance use	Semi-structured interviews with life grid; multiple services, organizations, and universities	A
Barnard (2003) Scotland	*N* = 36 (20F), 8–22	Parental drug use	Semi-structured interviews; treatment services/secure unit/rehabilitation unit	B
Bickelhaupt (2021) United States	*N* = 13 (9F), 21–25	Parental alcohol use	Semi-structured interviews; local state university; constant comparative analysis	A
Christensen (1997) Denmark	*N* = 32 (14F), 5–16	Parental alcohol use	Interviews; alcohol treatment institution for parents	C
D’Costa (2021) India	*N* = 15 (11F), 17–19	Parental alcohol use	Semi-structured interviews; treatment services for parents; thematic analysis	B
Dundas (2000) Norway	*N* = 17 (8F), 10–21	Parental alcohol use	Semi-structured interviews; out-patient clinic for parents’ alcohol use	C
Fraser (2009) England	*N* = 8 (4F), 4–14	Parental alcohol use	Draw and write semi-structured interviews; social services; phenomenological perspective	B
Hagström (2019) Sweden	*N* = 19 (8F), 6–24	Parental alcohol use	Longitudinal, three interviews over 13 years; children are people too program; narrative methods	A
M. Hill (1996) Scotland	*N* = 27, 5–12+	Parental alcohol use	Interviews; multiple agencies and services	C
L. Hill (2015) Scotland	*N* = 30 (16F), 9–20	Parental alcohol use	Group work, interviews, task-based activities; voluntary organizations; thematic analysis	C
Holmila (2011) Finland	*N* = 70 (58F), 12–18	Parental alcohol use	Online survey with open-ended questions; two websites for children with parental substance use; content analysis	C
Houmøller (2011) England (Bernays, 2011)	*N* = 50 (30F), 10–18	Parental substance use	Semi-structured interviews (16 young people had follow-up interviews over 20 months); specialist services for young people; thematic analysis	A
Johnson (2013) United States	*N* = 14 (6F), 14–17	Mother’s substance use	Semi-structured interviews; social services and schools; content analysis	B
McGuire (2002) Scotland	*N* = 7, Adolescence	Parental drug use	Semi-structured interviews; social work services and addiction treatment services	B
McLaughlin (2015) Northern Ireland	*N* = 23 (14F), 7–14	Parental alcohol use	Coproduction participatory workshops; pharos service at Barnardo’s; thematic analysis	B
Moore (2010) Australia (Moore, 2011)	*N* = 15 (8F), 11–17	Parental substance use	Semi-structured interviews with activities for engagement; services and organizations for young people; grounded theory	A
Mudau (2018) South Africa	*N* = 8 (4F), 14–25	Parental alcohol use	Interviews; local village and schools; thematic narrative analysis	C
Murray (1998) Canada	*N* = 5 (3F), 13–19	Parental alcohol use	Three interviews over 4 months; Al-Anon, school, personal contact; constant comparative analysis	A
Nattala (2020) India	*N* = 15 (10F), 10–19	Father’s alcohol use	Semi-structured interviews; outpatients for fathers in treatment and snowball sampling	A
O’Connor (2014) Wales	*N* = 13, 13–21	Parental substance use	Interviews; crisis intervention service (child protection register); thematic analysis	B
Offiong (2020) United States (Lewis, 2021; Powell, 2021)	*N* = 14 (6F), 18–24	Parental drug use	Semi-structured interviews; local organizations; content analysis	B
Park (2016) South Korea (Park, 2017; Park 2018)	*N* = 22 (14F), 19–30	Mainly father’s alcohol use	Two semi-structured interviews; two universities, one college, online self-help groups, siblings; thematic analysis	B
Ramirez (2014) Mexico	*N* = 4 (3F), 20–22	Father’s alcohol use	Life stories method with interview; one university; content analysis	C
Reupert (2012) Australia	*N* = 12 (6F), 8–15	Parental substance use	Semi-structured interviews; service for dual diagnosis families; interpretative phenomenological analysis	B
Ronel (2010) Israel (Ronel, 2011)	*N* = 19 (7F), 13–22	Parental substance use	Semi-structured interviews; treatment services for parents and services for young people; qualitative– constructivist method	B
Silva (2013) Brazil (Silva, 2013)	*N* = 40 (30F), 15–20	Parental alcohol use	Life history—semi-structured interviews; urban tribes project; thematic analysis	C
Tamutiené (2019) Lithuania	*N* = 23 (18F), 8–18	Parental alcohol use	Semi-structured interviews; social services; thematic analysis	C
Tinnfält (2011) Sweden	*N* = 27 (24F), 12–19	Parental alcohol use	Interviews/focus groups; support groups; content analysis	B
Tinnfält (2018) Sweden	*N* = 18 (8F), 7–9	Parental alcohol use	Interviews; treatment center for parents’ addiction; content analysis	A
Turning Point (2006) England/Wales	12–18	Parental alcohol use	Interviews; turning point services	B
Velleman (2008) England, Germany, Poland, Spain, and Malta (Templeton, 2009)	*N* = 48 (31F), 12–18	Parental alcohol use	Mixed method interview- standardized questionnaire with open ended questions (Alcohol Violence Teenager Version); treatment services for parents, support services for the young person; thematic analysis	C
Wangensteen (2019) Norway (Wangensteen & Westby, 2019; Wangensteen, 2020)	*N* = 12 (9F), 13–26	Parental substance use	Semi-structured interviews; treatment services for parents; interpretative phenomenological analysis	B
Yusay (2019) Philippines	*N* = 13 (10F), 13–19	Parental drug use	Interviews; community-based intervention program for parent’s substance use; narrative analysis	B

Four studies (six papers) reported on parental drug use only (Barnard & Barlow, 2003; Lewis et al., 2021; McGuire, 2002; Offiong et al., 2020; Powell et al., 2021; Yusay & Canoy, 2019). Ten studies (nineteen papers) focused on parental alcohol and/or drug use (Alexanderson & Näsman, 2017; Backett-Milburn et al., 2008; Bancroft et al., 2004; Bernays & Houmøller, 2011; Houmøller et al., 2011; Johnson, 2013; Moore et al., 2010, 2011; O’Connor et al., 2014; Reupert et al., 2012; Ronel & Haimoff-Ayali, 2010; Ronel & Levy-Cahana, 2011; Templeton et al., 2009; Velleman et al., 2008; Wangensteen et al., 2019, 2020; Wangensteen & Westby, 2019; Wilson et al., 2008, 2012). The remaining 21 studies (24 papers) primarily examined parental alcohol use. Four studies (five papers) focused on fathers’ use (Ahuja et al., 2003; Nattala et al., 2020; Park & Schepp, 2017; Park et al., 2016; Ramírez Dávila et al., 2014), one focused on mothers’ use (Johnson, 2013), while all remaining studies focused on substance use in either or both parents.

### Themes

Synthesis of 35 studies (49 papers) identified five overarching themes: (1) living with the unpredictable: insecurity within the family, (2) social and emotional impact of parental substance use, (3) controlling the uncontrollable: creating safety within the family, (4) coping with and resisting the emotional and social impacts, and (5) formal and informal support. Each theme also has a number of different sub-themes (see Supplemental Materials for a table documenting which studies are related to each theme and sub-theme).

### 1. Living With the Unpredictable: Insecurity Within the Family

#### Relationship with parent

The relationship between the child and parent who uses substances was often reported as unpredictable, described as a “never ending roller coaster” (Bickelhaupt et al., 2021, p. 7), with fluctuations in the levels of love and affection shown from the parent to the child. A minority voice within some studies included children and young people who reflected that their relationship with a parent who uses substances was not affected (Alexanderson & Näsman, 2017; Bancroft et al., 2004; Bernays & Houmøller, 2011; Fraser et al., 2009; M. Hill et al., 1996; Johnson, 2013; McGuire, 2002; Moore et al., 2011; Reupert et al., 2012; Silva et al., 2013; Tinnfält et al., 2018; Wilson et al., 2012), or even that they enjoyed the affection and generosity from their parents when they had been drinking alcohol (M. Hill et al., 1996). Others deemed such affection as “meaningless” (Bancroft et al., 2004, p. 12), and often described these relationships as being hostile and manipulative, with frequent arguments, tension, and conflict or less frequently reported as a “[roommate] kind of relationship” (Moore et al., 2010, p. 23).

#### Cycle of use

A common experience for the young people was the uncertainty that resulted from substance use fluctuation from abstinence to heavy use. Such fluctuation was reported to impact the unpredictable and chaotic nature of their parent’s behavior and mood, leading to inconsistent parenting. During periods of abstinence, most studies reported that young people experienced this as good and happy times, where they felt loved and cared for. Periods of substance use were viewed as stressful and scary, leading to issues of unsupervised care, neglect, and creating unsafe environments for children and young people. The unpredictable nature of not knowing when or if their parents would use substances again seemed to affect children and young people’s emotional well-being. During periods of lower use, young people could become anxious or worried about when their parent would drink or use drugs (Bancroft et al., 2004; Bernays & Houmøller, 2011; Fraser et al., 2009; Hagström & Forinder, 2019; L. Hill, 2015; Houmøller et al., 2011; Moore et al., 2011; Nattala et al., 2020; Park & Schepp, 2017; Tinnfält et al., 2018; Wangensteen et al., 2019, 2020). In Moore et al. (2011), a 17-year-old male stated that, “there were the frantic times, when there were weeks when it was worse, or weeks when it seemed completely normal. I would start looking out for stuff during these good times” (p. 167). Younger children were described as having hope that their parents had stopped for good while older children recalled “losing hope” after witnessing several failed attempts by their parents to stop. However, these children began to predict the unpredictable, and were better able to find a path through the insecurity (Alexanderson & Näsman, 2017; Bancroft et al., 2004; Christensen, 1997; D’Costa & Lavalekar, 2021; Moore et al., 2010; Silva & Padilha, 2013; Yusay & Canoy, 2019).

#### Roles and responsibilities

A further common theme within the literature was the caring responsibilities that children and young people had taken on for other members of their family, which felt unpredictable when parents stopped use and took back the parental role from children (Ahuja et al., 2003; Backett-Milburn et al., 2008; Bancroft et al., 2004; Bernays & Houmøller, 2011; D’Costa & Lavalekar, 2021; Fraser et al., 2009; Hagström & Forinder, 2019; M. Hill et al., 1996; Holmila, Itäpuisto, & Ilva, 2011; Houmøller et al., 2011; Johnson, 2013; Lewis et al., 2021; McGuire, 2002; McLaughlin et al., 2015; Moore et al., 2010, 2011; Murray, 1998; Nattala et al., 2020; O’Connor et al., 2014; Offiong et al., 2020; Park et al., 2016; Ramírez Dávila et al., 2014; Reupert et al., 2012; Ronel & Haimoff-Ayali, 2010; Ronel & Levy-Cahana, 2011; Templeton et al., 2009; Turning Point, 2006). These relationships often resulted in the blurring of roles between being a child, sister, or brother and being a parent to siblings or parents. Such unpredictability led to confusion, tension, and arguments within the family, with young people viewing family members as lacking in care and support or finding it hard to relinquish these roles (Alexanderson & Näsman, 2017; Backett-Milburn et al., 2008; Bancroft et al., 2004; Hagström & Forinder, 2019; Holmila et al., 2011; Johnson, 2013; Moore et al., 2011; Murray, 1998; Park et al., 2016; Ramírez Dávila et al., 2014; Ronel & Haimoff-Ayali, 2010; Turning Point, 2006). In Bancroft et al. (2004), a 17-year-old female reflected on this experience, “I’m used tae daen (to doing) all the tidying and the cooking and like telling [siblings] when tae be in . . . And my mum’s started daen that and . . . it’s like a kind of conflict between us now” (p. 10).

#### Living arrangements

The lack of stability within their living arrangements and home environment played into the experience of insecurity for children and young people. Young people recalled having transient lifestyles, with frequent moves, often described as chaotic, leading to young people feeling unsettled (Alexanderson & Näsman, 2017; Backett-Milburn et al., 2008; Bancroft et al., 2004; Fraser et al., 2009; L. Hill, 2015; Houmøller et al., 2011; Lewis et al., 2021; McGuire, 2002; Moore et al., 2010, 2011; O’Connor et al., 2014; Offiong et al., 2020; Park et al., 2016; Reupert et al., 2012; Ronel & Haimoff-Ayali, 2010; Tamutienė & Jogaitė, 2019; Templeton et al., 2009; Turning Point, 2006; Wangensteen & Westby, 2019; Wilson et al., 2008). A 10-year-old female recalled her experience of such transience, “I use to live with my mum, but she got a bit ill, so we moved into Gran’s house. Then she got better (sighs), so we moved back down, and then she got a bit ill again, and then she got better . . . That was a big breath! Phew” (L. Hill, 2015, p. 348). Furthermore, some young people also experienced the stress and insecurity of the often-present threat that they would be forced to leave the family home by a parent (Ahuja et al., 2003; Backett-Milburn et al., 2008; Johnson, 2013; Lewis et al., 2021; Nattala et al., 2020; Wilson et al., 2008). When recalling the home environment, some children and young people described it as untidy, unstable and one in which “unsafe adults” frequently visited (Backett-Milburn et al., 2008; Bancroft et al., 2004; Hagström & Forinder, 2019; Houmøller et al., 2011; McGuire, 2002; Moore et al., 2010; Murray, 1998; Park & Schepp, 2018; Park et al., 2016; Reupert et al., 2012; Wangensteen & Westby, 2019). Regardless of their parent’s substance use and subsequent insecurity, many children perceived family as important, felt a strong loyalty to their parents, and wanted to belong to a family (Alexanderson & Näsman, 2017; Backett-Milburn et al., 2008; Bernays & Houmøller, 2011; Dundas, 2000; Houmøller et al., 2011; Moore et al., 2011; Reupert et al., 2012; Tinnfält et al., 2018; Turning Point, 2006; Wangensteen & Westby, 2019; Wilson et al., 2012). Where young people did not have close family relationships, they spoke about developing family-like relationships with others, including friends, social workers, or teachers (Backett-Milburn et al., 2008; Bancroft et al., 2004; McLaughlin et al., 2015; O’Connor et al., 2014; Offiong et al., 2020; Wilson et al., 2008, 2012).

### 2. Social and Emotional Impact of Parental Substance Use

#### Family adversity

Children and young people commonly experienced interrelating and compounding factors beyond parental substance use, which contributed to the complexity, insecurity, and trauma within children and young people’s lives. These cumulative factors led to one 23-year-old male recounting his experiences as, “the most hellish experience that you could ever imagine” (Backett-Milburn et al., 2008, p. 466). Across most studies, many young people were additionally exposed to parental intimate partner violence and abuse (IPVA), violence and abuse against them directly, siblings or pets, as well as parental mental health problems, intergenerational substance use, or family imprisonment. A minority of young people also recalled incidents when parents forced them to use substances (Alexanderson & Näsman, 2017; Hagström & Forinder, 2019; Nattala et al., 2020). IPVA compounded their difficult situation and was associated with feelings of abandonment and a lack of protection (Alexanderson & Näsman, 2017). However, children and young people spoke more about the harmful impact of parental alcohol use than violence in their families (Templeton et al., 2009), while others perceived parental mental health problems to have a particularly detrimental impact on them (Bancroft et al., 2004).

#### Emotional impacts

The emotional impacts of living with parental substance use and other adverse childhood experiences, were reported in all studies. Children and young people often reported experiencing mental health problems and feeling “hurt in the inside” (M. Hill et al., 1996, p. 163), including feelings of sadness and depression, fear, anxiety, and worry as well as describing externalized feelings of anger that “erupt like a volcano” (McLaughlin et al., 2015, p. 46). A minority of young people experienced guilt or blame for their parent’s substance use at a young age, before they realized they were not to blame (Bickelhaupt et al., 2021; Christensen, 1997; M. Hill et al., 1996; Mudau, 2018; Murray, 1998; Park & Schepp, 2017; Turning Point, 2006). Additionally, it was often reported that caring responsibilities within the family felt burdensome, whereby young people expressed a sense of loss at not having a normative childhood, missed opportunities for family bonding, and decreased self-esteem and confidence as they abandoned their own needs for the needs of their families. Yet, a minority of young people described such roles as improving their self-esteem (Backett-Milburn et al., 2008; Bancroft et al., 2004; D’Costa & Lavalekar, 2021; O’Connor et al., 2014; Ronel & Haimoff-Ayali, 2010). Siblings also tended to experience and be impacted by parental substance use differently, depending on birth order. Younger siblings often reported being protected or shielded by their older siblings but became more vulnerable if their older siblings subsequently left home. Older siblings had advanced understandings of parental substance use but opportunity for independence and space (Alexanderson & Näsman, 2017; Backett-Milburn et al., 2008; Bancroft et al., 2004; Bernays & Houmøller, 2011; Houmøller et al., 2011; Templeton et al., 2009). Children and young people also reported experiencing low confidence, poor self-esteem, and limited hope for the future (Moore et al., 2010, 2011; Murray, 1998; Nattala et al., 2020; Park et al., 2016; Ronel & Levy-Cahana, 2011). Such emotional distress was described as affecting some children and young people’s physical health, sleep, and diet (Bickelhaupt et al., 2021; Hagström & Forinder, 2019; Holmila et al., 2011; Houmøller et al., 2011; Nattala et al., 2020; Templeton et al., 2009; Velleman et al., 2008).

#### Stigma and shame

Young people were often impacted by the secrecy of substance use within the family, whereby parents’ continued efforts to hide, disguise, or deny their substance use established the topic as taboo, and created the perception that substance use is embarrassing, shameful, and to be hidden (Backett-Milburn et al., 2008; Barnard & Barlow, 2003; Houmøller et al., 2011). Young people reported feeling great shame and embarrassment when they realized that their families were unlike other families, and that their parent’s behavior was not perceived as “normal” within society. Such induced shame due to the association with parental substance use led to fear of being treated unfairly (Bancroft et al., 2004; Barnard & Barlow, 2003; Bernays & Houmøller, 2011; Christensen, 1997; Dundas, 2000; Holmila et al., 2011; Houmøller et al., 2011; McGuire, 2002; Park et al., 2016) or judged and rejected by others regardless of enacted discrimination (Backett-Milburn et al., 2008; Bancroft et al., 2004; Holmila et al., 2011; Houmøller et al., 2011; McGuire, 2002; Moore et al., 2010; Mudau, 2018; Murray, 1998; Yusay & Canoy, 2019). However, if others found out about parental substance use, stigma, bullying, and discrimination often ensued (Backett-Milburn et al., 2008; Bancroft et al., 2004; Barnard & Barlow, 2003; Bernays & Houmøller, 2011; Fraser et al., 2009; Hagström & Forinder, 2019; M. Hill et al., 1996; Houmøller et al., 2011; McGuire, 2002; Moore et al., 2010; Nattala et al., 2020; O’Connor et al., 2014; Tamutienė & Jogaitė, 2019; Tinnfält et al., 2018; Wangensteen et al., 2020). Experiencing shame, stigma, and discrimination impacted on young people’s emotional development, their ability to trust and develop social relationships, and perpetuated the isolation felt by young people (Bancroft et al., 2004; Hagström & Forinder, 2019; Houmøller et al., 2011; McGuire, 2002; Moore et al., 2010; Mudau, 2018; Nattala et al., 2020; Offiong et al., 2020; Reupert et al., 2012; Tamutienė & Jogaitė, 2019; Turning Point, 2006; Yusay & Canoy, 2019).

#### Poverty and financial impact

Many young people had been exposed to poverty throughout their lives, with resources further diminished by parental substance use. Exposure to poverty and the financial impact of parental substance use left little money for things such as food, clean clothes, or school fees (Houmøller et al., 2011; McGuire, 2002; Moore et al., 2010; Mudau, 2018; Nattala et al., 2020; Ramírez Dávila et al., 2014; Yusay & Canoy, 2019), and reportedly resulted in some young people feeling shame as well as being bullied (Houmøller et al., 2011; McGuire, 2002; Park & Schepp, 2018; Tamutienė & Jogaitė, 2019). While one study found that children of substance using parents experienced stigma regardless of their socioeconomic status (Hagström & Forinder, 2019), other studies reported a socioeconomic advantage from belonging to a higher social class or lack of exposure to poverty (Bancroft et al., 2004; McGuire, 2002; Ronel & Levy-Cahana, 2011). Within these families, parents could purchase lifestyles which were relatively free of discrimination and stigma relating to their alcohol or drug use, as they could more easily hide it from others. A young person recalled their reasons for not being bullied was because they, “always had the best of gear [clothes]” (McGuire, 2002, p. 26).

### 3. Controlling the Uncontrollable: Creating Safety Within the Family

#### Agency and safety

While young people were generally negatively impacted by parental substance use, they were not passive within these experiences and often reported trying to “control the situation” at home or within their family (D’Costa & Lavalekar, 2021, p. 20). Hypervigilance allowed children and young people to notice signs and clues that better prepared them for escalating substance use, imminent conflict, violence, or abuse (Backett-Milburn et al., 2008; Barnard & Barlow, 2003; Bernays & Houmøller, 2011; Bickelhaupt et al., 2021; Christensen, 1997; Fraser et al., 2009; Hagström & Forinder, 2019; L. Hill, 2015; M. Hill et al., 1996; Houmøller et al., 2011; McGuire, 2002; Moore et al., 2011; Tinnfält et al., 2018; Velleman et al., 2008). Being able to identify potentially risky situations allowed young people to adapt, mediate, control, or avoid such escalating situations, keeping them safe and able to survive. Children and young people spoke of ways they enacted agency by taking control of their environment and creating safe spaces for themselves and siblings to escape within an otherwise unsafe home (Ahuja et al., 2003; Backett-Milburn et al., 2008; Bancroft et al., 2004; Bickelhaupt et al., 2021; Christensen, 1997; D’Costa & Lavalekar, 2021; Dundas, 2000; Hagström & Forinder, 2019; M. Hill et al., 1996; Holmila et al., 2011; Houmøller et al., 2011; Johnson, 2013; Nattala et al., 2020; Park & Schepp, 2017, 2018; Park et al., 2016; Ramírez Dávila et al., 2014; Templeton et al., 2009; Tinnfält et al., 2018; Turning Point, 2006; Velleman et al., 2008; Wangensteen et al., 2019; Yusay & Canoy, 2019). In Hagström and Forinder (2019), a 6-year-old boy would, “hide in a small space under the house with a torch” as it was “a scary dark place where no one else dares to go” (p. 16). This allowed children and young people to resist parents’ threatening and controlling behaviors by finding ways to minimize contact with the parent, for example, by taking up hobbies or spending extended periods of time at the homes of others (Ahuja et al., 2003; Alexanderson & Näsman, 2017; Backett-Milburn et al., 2008; Bancroft et al., 2004; Dundas, 2000; Hagström & Forinder, 2019; Holmila et al., 2011; Houmøller et al., 2011; Johnson, 2013; McGuire, 2002; Moore et al., 2010, 2011; Nattala et al., 2020; O’Connor et al., 2014; Park & Schepp, 2017; Reupert et al., 2012; Ronel & Haimoff-Ayali, 2010; Templeton et al., 2009; Tinnfält et al., 2018; Turning Point, 2006; Velleman et al., 2008; Wangensteen & Westby, 2019; Wilson et al., 2012). They also constantly monitored their parent’s reactions, trying to understand their parent’s emotions, and adapted their response to the perceived mood (Bernays & Houmøller, 2011; D’Costa & Lavalekar, 2021; Dundas, 2000; Hagström & Forinder, 2019; Park & Schepp, 2017; Park et al., 2016; Reupert et al., 2012; Tinnfält et al., 2018; Yusay & Canoy, 2019). In Reupert et al. (2012), an 8-year-old boy recalled, “It’s important that I am good and [do] not make dad angry” (p. 157). Additionally, gaining independence from the family allowed young people a sense of control over their relationships and to put their needs first (Backett-Milburn et al., 2008; Bancroft et al., 2004; Bernays & Houmøller, 2011; Bickelhaupt et al., 2021; Hagström & Forinder, 2019; Houmøller et al., 2011; Park & Schepp, 2017; Ramírez Dávila et al., 2014; Ronel & Haimoff-Ayali, 2010; Wangensteen et al., 2019; Wilson et al., 2012). However, it was difficult for some children and young people to fully gain independence from these relationships (Ahuja et al., 2003; Backett-Milburn et al., 2008; Bancroft et al., 2004; Houmøller et al., 2011; Wilson et al., 2012), even more so for young people living in societies where cultural norms expected children to support their aging parents (Park et al., 2016).

#### Controlling parental substance use and conflict

Some children described trying to control their parent’s substance use by hiding or throwing away substances or hiding money (Ahuja et al., 2003; Backett-Milburn et al., 2008; Bancroft et al., 2004; D’Costa & Lavalekar, 2021; Fraser et al., 2009; Hagström & Forinder, 2019; M. Hill et al., 1996; Moore et al., 2011; Nattala et al., 2020; Tinnfält et al., 2018). As they aged and gained power, in terms of physical, relational, and emotional strength, young people reported mediating conflict, by putting themselves in harm’s way to protect their non-using parent or siblings and to defuse escalating arguments (Ahuja et al., 2003; Alexanderson & Näsman, 2017; Bancroft et al., 2004; Barnard & Barlow, 2003; Bernays & Houmøller, 2011; D’Costa & Lavalekar, 2021; Hagström & Forinder, 2019; M. Hill et al., 1996; Holmila et al., 2011; Houmøller et al., 2011; Johnson, 2013; McGuire, 2002; Moore et al., 2011; Nattala et al., 2020; Park & Schepp, 2018; Park et al., 2016; Ramírez Dávila et al., 2014; Ronel & Haimoff-Ayali, 2010; Silva & Padilha, 2013; Templeton et al., 2009; Tinnfält et al., 2018; Velleman et al., 2008). Some also tried to confront their parent about substance use or gave ultimatums (Backett-Milburn et al., 2008; Bancroft et al., 2004; Christensen, 1997; Hagström & Forinder, 2019; Holmila et al., 2011; Johnson, 2013; McGuire, 2002; McLaughlin et al., 2015; Nattala et al., 2020; Park & Schepp, 2017; Templeton et al., 2009; Turning Point, 2006; Yusay & Canoy, 2019).

To avoid conflict between their parents, some young people recalled withholding information about their experiences from their non-using parent (Alexanderson & Näsman, 2017; Dundas, 2000; Hagström & Forinder, 2019; Johnson, 2013; Park et al., 2016; Turning Point, 2006) or more rarely, by contacting services, for example, police or social care, to help diffuse situations (Holmila et al., 2011; Tamutienė & Jogaitė, 2019). Where they could, young people reported trying to avoid putting themselves into danger when they lived between separated parents, by calling to see if their parent was sober before returning home (Alexanderson & Näsman, 2017; Hagström & Forinder, 2019). Trying to control escalating situations between their parents with context-specific expertize, and negotiating the boundaries between risk and safety, were intended to get themselves or others out of harm’s way. However, some experienced repercussions, in terms of violence toward them or their family (Ahuja et al., 2003; Alexanderson & Näsman, 2017; Backett-Milburn et al., 2008; Bancroft et al., 2004; M. Hill et al., 1996; Moore et al., 2010; Mudau, 2018; Nattala et al., 2020; Powell et al., 2021; Ramírez Dávila et al., 2014).

### 4. Coping With and Resisting the Emotional and Social Impacts

#### Coping with the emotional impacts

Children and young people reported seeking to resist the emotional impacts of parental substance use by writing in journals, practicing mindfulness, or taking part in fun activities (D’Costa & Lavalekar, 2021; Dundas, 2000; Hagström & Forinder, 2019; Holmila et al., 2011; Tinnfält et al., 2018; Velleman et al., 2008). More passive strategies used to cope, for example, avoiding thinking about their circumstances, reportedly had negative consequences on their mental health (Backett-Milburn et al., 2008; Bickelhaupt et al., 2021). Other young people externalized their emotions through anti-social behaviors including violence and bullying, offending, or substance use (Ahuja et al., 2003; Alexanderson & Näsman, 2017; Backett-Milburn et al., 2008; Bancroft et al., 2004; Barnard & Barlow, 2003; Bickelhaupt et al., 2021; Fraser et al., 2009; Hagström & Forinder, 2019; L. Hill, 2015; Holmila et al., 2011; Lewis et al., 2021; Moore et al., 2010; Murray, 1998; O’Connor et al., 2014; Offiong et al., 2020; Park et al., 2016; Ronel & Haimoff-Ayali, 2010; Ronel & Levy-Cahana, 2011; Tamutienė & Jogaitė, 2019; Templeton et al., 2009; Tinnfält, Eriksson, & Brunnberg, 2011; Tinnfält et al., 2018; Turning Point, 2006; Wilson et al., 2008). Some young people also reported self-harming behaviors to cope with the emotional impact (Bickelhaupt et al., 2021; Holmila et al., 2011; Nattala et al., 2020; Tamutienė & Jogaitė, 2019; Velleman et al., 2008). In Tamutienė and Jogaitė (2019), a 17-year-old female reflected on her experiences of how her externalized behaviors showed emotional impact as well as a call for help that she did not receive, when she, “stopped attending classes, started talking to teachers harshly and later started self-harming.” “I was showing how bad it was for me, and later, I started consuming alcohol and drugs at school” (p. 215).

#### Resisting the social impacts

The majority of children and young people made efforts to hide their parents’ substance use in order to reportedly resist the social impacts of parental substance use, including stigma, embarrassment, and fear of endangering social relationships (Backett-Milburn et al., 2008; Bancroft et al., 2004; Barnard & Barlow, 2003; Bernays & Houmøller, 2011; Christensen, 1997; D’Costa & Lavalekar, 2021; Hagström & Forinder, 2019; M. Hill et al., 1996; Holmila et al., 2011; Houmøller et al., 2011; McGuire, 2002; Moore et al., 2010; Murray, 1998; Nattala et al., 2020; Park & Schepp, 2018; Park et al., 2016; Reupert et al., 2012; Tamutienė & Jogaitė, 2019; Templeton et al., 2009; Tinnfält et al., 2011; Tinnfält et al., 2018; Turning Point, 2006; Velleman et al., 2008; Wangensteen et al., 2020; Wilson et al., 2008; Yusay & Canoy, 2019). An 18-year-old female recounted her reasons for non-disclosure, “I didn’t really like to talk to my friends about it . . . it was embarrassing, who wants to admit their families are alkies?” (Turning Point, 2006, p. 12). For some young people, the experience of parental drug use was seen as more stigmatizing and embarrassing to disclose than parental alcohol use (Barnard & Barlow, 2003). Other less-cited reasons for choosing not to disclose included fear of removal from the family, fear of repercussions for the parent or being disloyal, and fear of violent repercussions. Conversely, some young people also enacted agency by choosing to tell someone about their parent’s substance use, sometimes but not always, with favorable supportive outcomes (Alexanderson & Näsman, 2017; Backett-Milburn et al., 2008; Bancroft et al., 2004; Bernays & Houmøller, 2011; Christensen, 1997; D’Costa & Lavalekar, 2021; Hagström & Forinder, 2019; L. Hill, 2015; Holmila et al., 2011; Houmøller et al., 2011; Johnson, 2013; McGuire, 2002; McLaughlin et al., 2015; Moore et al., 2010; Mudau, 2018; Nattala et al., 2020; Park & Schepp, 2018; Powell et al., 2021; Tamutienė & Jogaitė, 2019; Templeton et al., 2009; Tinnfält et al., 2011; Tinnfält et al., 2018; Turning Point, 2006; Velleman et al., 2008; Wangensteen et al., 2019; Wilson et al., 2012). Moreover, younger children did not always choose to speak to people but enacted small gestures of defiance to their parents’ hidden use by talking to pets or toys (Hagström & Forinder, 2019; Holmila et al., 2011; McLaughlin et al., 2015), for example, a 6-year-old-boy stated, “I talk to the bird. She’s a friend. I tell my secret to the bird. I only whisper it to her” (Hagström & Forinder, 2019, p. 17). While young people were finding ways to show resistance, it also highlighted their isolated and lonely position.

### 5. Formal and Informal Support

#### Sources of support

Emotional and social support were mainly cited as being provided by older siblings, a non-using parent, an extended family member, friend, or neighbor (Alexanderson & Näsman, 2017; Backett-Milburn et al., 2008; Bancroft et al., 2004; Bernays & Houmøller, 2011; D’Costa & Lavalekar, 2021; Dundas, 2000; Hagström & Forinder, 2019; L. Hill, 2015; M. Hill et al., 1996; Holmila et al., 2011; Houmøller et al., 2011; Johnson, 2013; Lewis et al., 2021; McGuire, 2002; McLaughlin et al., 2015; Mudau, 2018; Nattala et al., 2020; O’Connor et al., 2014; Offiong et al., 2020). However, these forms of informal support were not always accessible, long-lasting, or safe, as some of these relationships were seen as inducing further risk to the young person, especially friends who encouraged substance use and offending behaviors (Backett-Milburn et al., 2008; Bancroft et al., 2004; McGuire, 2002; Ronel & Haimoff-Ayali, 2010; Ronel & Levy-Cahana, 2011; Tamutienė & Jogaitė, 2019; Wilson et al., 2008). Less often, young people reflected on the formal support they had received from within the healthcare, social care, and education systems that reportedly provided both help and hindrance (Backett-Milburn et al., 2008; Bancroft et al., 2004; Bernays & Houmøller, 2011; Fraser et al., 2009; Houmøller et al., 2011; Johnson, 2013; McGuire, 2002; McLaughlin et al., 2015; Moore et al., 2010; O’Connor et al., 2014; Offiong et al., 2020; Powell et al., 2021; Tamutienė & Jogaitė, 2019; Tinnfält et al., 2011; Turning Point, 2006; Wangensteen & Westby, 2019; Wilson et al., 2008, 2012).

Within both formal and informal forms of support, children and young people viewed interactions that were genuine, caring, compassionate, and non-stigmatizing, as helping them to feel safe and trust the other person. To build these relationships, young people spoke of needing time, consistency, flexibility, and “the need for someone stable” (Offiong et al., 2020, p. 4). Within formal forms of support provision, it was the informal approach that was often seen as most useful, for instance, a head teacher who allowed a young person who was having a difficult day to “sit in a corner on a beanbag and work in her office” and to “have a cup of tea and a biscuit” (Houmøller et al., 2011, p. 59). However, children and young people also reflected that the quality of the relationship could be detrimental to support provision when the opposite occurred, including lack of trust, lack of consistency due to high turnover of staff, rigidity in the support provided, and feeling like they are being pressured for information. Further, some young people had experienced stigma and prejudice from professionals within education (Backett-Milburn et al., 2008; Bancroft et al., 2004; McGuire, 2002; Nattala et al., 2020; Tamutienė & Jogaitė, 2019; Wilson et al., 2008), social care (McGuire, 2002), healthcare (Hagström & Forinder, 2019) or from a range of practitioners in the health, care, and education system (Wangensteen et al., 2020), impacting the support they received. Moreover, young people stated that the lack of action or adequate action when disclosure occurred left them feeling abandoned and less likely to seek further support (Bancroft et al., 2004; Hagström & Forinder, 2019; Houmøller et al., 2011; Tamutienė & Jogaitė, 2019; Templeton et al., 2009; Tinnfält et al., 2011; Turning Point, 2006; Velleman et al., 2008; Wangensteen et al., 2019). Some young people also recalled times when they did not meet the eligibility criteria or age restrictions for support, leaving them further isolated (Moore et al., 2010; Offiong et al., 2020; Wilson et al., 2008).

#### School environment

School was frequently cited within studies, often viewed by young people as a place of safety and support, but not without risk. Primary school was reported as a place for young people to see friends, explore hobbies, and have time for them away from concerns at home (D’Costa & Lavalekar, 2021; McLaughlin et al., 2015). However, problems tended to arise at secondary school where it became a place to worry about home, often leading to young people skipping school (Backett-Milburn et al., 2008; Barnard & Barlow, 2003; Dundas, 2000; Hagström & Forinder, 2019; L. Hill, 2015; Lewis et al., 2021; Moore et al., 2010; Nattala et al., 2020; O’Connor et al., 2014; Turning Point, 2006) or struggling to keep up with their schoolwork (Holmila et al., 2011; Moore et al., 2010; Mudau, 2018; Nattala et al., 2020; Park & Schepp, 2017; Templeton et al., 2009; Turning Point, 2006). Achieving and doing well at school was viewed as a useful strategy to lead a successful life (Ahuja et al., 2003; Bancroft et al., 2004; Bickelhaupt et al., 2021; D’Costa & Lavalekar, 2021; Hagström & Forinder, 2019; M. Hill et al., 1996; Houmøller et al., 2011; Nattala et al., 2020; Park & Schepp, 2017, 2018; Ramírez Dávila et al., 2014; Ronel & Haimoff-Ayali, 2010; Ronel & Levy-Cahana, 2011; Turning Point, 2006; Wangensteen & Westby, 2019; Wilson et al., 2008). However, this was not always easy, due to some young people being excluded or suspended for their unacceptable behavior, further isolating them from social and professional support (Bancroft et al., 2004; Tamutienė & Jogaitė, 2019; Turning Point, 2006; Wilson et al., 2008). Young people reported wanting school staff to recognize the impacts of parental substance use on children, to improve referral and early access to support (Hagström & Forinder, 2019; Holmila et al., 2011; Moore et al., 2011; Tamutienė & Jogaitė, 2019; Tinnfält et al., 2011; Turning Point, 2006). While externalized behaviors were reported as being easier to identify, this was not always the case for internalized feelings such as anxiety or fear, due to some pretending that everything was okay, to not incur social stigma (Bernays & Houmøller, 2011; D’Costa & Lavalekar, 2021; Houmøller et al., 2011; Tinnfält et al., 2011). A young person reflected, “even though I was having them problems at home I didn’t let it show in school. I’d still come in and do my work and act like a normal kid” (Houmøller et al., 2011, p. 28).

#### (Un)helpful helping

The focus of services on supporting the parent and ignoring the needs of the child was reportedly experienced negatively by young people as they wanted support for themselves (Alexanderson & Näsman, 2017; Moore et al., 2010; Tamutienė & Jogaitė, 2019; Wangensteen et al., 2019). A 21-year-old male expressed that “people keep talking about my mother: Your mum is on drugs, your mum is off drugs, your mum is in treatment . . . I do understand it, but we never talked much about me” (Wangensteen et al., 2019, p. 205). Support that included the whole family was viewed as useful when it alleviated family stress and conflict or improved family connectedness (Moore et al., 2010, 2011; Reupert et al., 2012; Tinnfält et al., 2018) but it was hard to talk openly in front of parents (Bancroft et al., 2004). Other young people wanted to have family support that focused on members of the family separately but concurrently (Moore et al., 2010). Kinship care was usually viewed positively (Bancroft et al., 2004; Fraser et al., 2009; L. Hill, 2015; Lewis et al., 2021), but did not always solve the emotional impact (Christensen, 1997). Young people wanted practical and financial aid to support the family (Moore et al., 2010, 2011; Park & Schepp, 2018; Powell et al., 2021; Reupert et al., 2012; Tamutienė & Jogaitė, 2019; Templeton et al., 2009; Velleman et al., 2008) or substance support for their parents alongside their own emotional support (Christensen, 1997; Holmila et al., 2011; McGuire, 2002; Moore et al., 2010; Reupert et al., 2012). Understanding more about substance use was viewed as useful and was sometimes searched for online (Bernays & Houmøller, 2011; Bickelhaupt et al., 2021; D’Costa & Lavalekar, 2021; Houmøller et al., 2011; Johnson, 2013; Murray, 1998; O’Connor et al., 2014; Park & Schepp, 2017, 2018; Turning Point, 2006; Velleman et al., 2008; Wangensteen et al., 2019; Wangensteen et al., 2020). Being involved in religious communities (D’Costa & Lavalekar, 2021; M. Hill et al., 1996; McLaughlin et al., 2015; Nattala et al., 2020) or meeting with those in similar situations were also sources of useful support (Bancroft et al., 2004; L. Hill, 2015; M. Hill et al., 1996; Holmila et al., 2011; McLaughlin et al., 2015; Moore et al., 2011; Moore et al., 2010; Mudau, 2018; Powell et al., 2021; Reupert et al., 2012; Tinnfält et al., 2011; Turning Point, 2006; Velleman et al., 2008).

## Discussion

This evidence synthesis of qualitative literature focused on children and young people’s coping strategies, perceived impact, and experiences of parental substance use (see Table 2 for an overview). These children and young people reported living highly disrupted and chaotic lives, characterized by unpredictability and insecurity within their familial relationships. It was also evident that children and young people often experienced co-occurring or clustering of childhood adversities, including poverty. Adverse childhood experiences are potentially traumatic events that can have negative and persistent child and adult health outcomes (Felitti et al., 1998). In a recent study based on UK longitudinal data, Adjei et al. (2022) found that poverty can amplify children’s experiences of adversities and is strongly associated with adverse child outcomes later in adolescence, including poor mental health. This review also found that lived experiences of insecurity and adversity reportedly impacted children and young people emotionally and socially, with resulting mental health problems, stigma, isolation, and loneliness. Children and young people often continued to experience emotional distress, even when their parents had stopped using substances or during times of abstinence, showing that these times can feel unsafe too due to dread of resumption. This finding identifies a need for ongoing emotional support for children, due to the recurrent nature of addiction. Focusing on removing risk only, by reducing parental substance use, neglects how the young person is feeling and may lead to worsening of outcomes. Services are not often structured to provide ongoing support, due mainly to reduced funding, that can be flexible to meet the needs of the child.

**Table 2.** Critical Findings.

From a thematic synthesis of 35 studies, covering the views of over 700 children and young people across twenty countries, we identified five themes in the qualitative literature for those that experienced parental alcohol and/or drug use. These included the major themes of children and young people’s control within the family to create safety, and enacting agency, and resistance to cope with the social and emotional impacts of parental substance use.

Most of the included studies reported the negative impacts of parental substance, without recognizing young people’s agency and attempts to change, control, and resist their experiences or impacts. Within a similar field, Arai et al. (2021) conducted a qualitative systematic review of young people’s experiences of IPVA and found comparable themes on children’s agency and coping, whereby children found creative and meaningful ways to change their situations. While such strategies demonstrate resilience, this is often in ways that place them in danger or that receive sanctions in society (e.g., externalizing behaviors), and therefore can increase rather than decrease vulnerability. Child-focused interventions need to support children and young people whose parents use substances to be agentic while also increasing their emotional and social resilience. To develop agency and resilience among children and young people who have experienced IPVA, Fellin et al. (2019) proposed a group-based intervention that builds on the strengths and skills young people have developed during their experiences, including strategies to build a sense of safety, develop trust in themselves and others and, build positive self-identity; similar strategies could be explored for children within the context of parental substance use.

Children and young people reported experience of shame, stigma, and discrimination due to their close association and relationship with a parent who uses substances, which was further compounded for those who had experienced poverty or lower socioeconomic status. Kotova (2020), proposed a multifaceted and cumulative model of stigmatization that considers such associative stigma as well as stigma associated with socially excluded backgrounds of families of people in prison, which was amplified by political, legal, and social views about value and worth. Similarly, some young people whose parents use substances move from a position of being stigmatized due to their association with parental substance use, and their socially excluded backgrounds, to direct stigma and discrimination due to their own externalized behaviors, without recognition of their lived experience and trauma (Muir et al., 2022). Within the United Kingdom, the construction and labeling of some families and young people as “troubled” due, in part, to substance use, has been driven by government policies, national programs and media depictions, many of which have been viewed as propagating stigma (Cameron, 2011; Goldson & Muncie, 2015). Moreover, children and young people are often encouraged to strive for a version of success in a society that platforms achievement at school, full employment, and a stable family. However, this pathway to success may be problematic for those who are having to navigate stigma in a system intent on reproducing structures of inequality. Those young people who are seen in practice and policy as “risky” (Bancroft & Wilson, 2007), due to their own substance use or offending may be trying to cope with the impacts of parental substance use, but because their form of coping is stigmatized, they are likely to experience discriminatory interactions and further negative outcomes. Practitioners need to be mindful of how stigma can exacerbate experiences for young people, including discriminatory behaviors within their own practice, especially to those who display externalized behaviors.

Most children and young people reported relying on informal forms of support rather than formal support. Yet, extended family members, siblings or peers are not always accessible or reliable due to the temporary or fluctuating nature of such relationships and may not be the best option for young people to provide ongoing support. It also places the burden of support on those who may also be exposed to substance use. The quality of the relationship of the person who was providing the support to the child was seen as important. Relational practice is about building an understanding of children and young people’s lived experience; establishing trust-based and respectful relationships; as well supporting them to be at the center of decision-making processes, which is often and increasingly seen in social care work (Ferguson et al., 2022; Munford, 2022). Similar relationship-building practices can also be seen within trauma-informed care, whereby any professional presumes all those they encounter have experienced trauma in some way and at some point, enabling supportive, nurturing, and non-stigmatizing relationships from the onset (Goddard, 2021). Such relationship-building practices and trauma-informed responses should be implemented within practice and included in interventions for children.

Evidence-based interventions in this arena are still largely focused on the people using substances themselves (McGovern, Newham, et al., 2021; McGovern, Smart, et al., 2021) or on family-based approaches (Templeton et al., 2010). Therefore, there appears to be a need for child-focused interventions that are effective, feasible, and relevant, as most current interventions show mixed or low-quality effect (McGovern, Smart, et al., 2021). A limited number of studies in this review considered from a young person’s point of view what support or resources would be most helpful to improve emotional and social well-being. There was also no evidence that considered how to reduce stigma. A systematic review exploring the effectiveness of interventions for reducing substance-related stigma could highlight useful strategies for those exposed to someone else’s substance use (Livingston et al., 2012). Group-based therapies were useful for self-stigma and shame. Communicating positive and inspirational stories to the wider public was useful for social stigma. For stigma at the structural level, training and educational programs were effective. However, interventions that counter-act the stigma, shame, and impacts that young people face, may not be meaningful if policies for childhood socioeconomic conditions such as poverty are not also considered. Additionally, it is important to find better, more relevant, and accessible strategies to help young people. Utilizing coproduction methods, whereby relevant stakeholders form a partnership with researchers to collaborate on all aspects of intervention development (Boyd et al., 2012) provides insights into feasibility, leading to context-specific, acceptable, and sustainable interventions within the community (Cargo & Mercer, 2008; Davies et al., 2015). Coproducing resources alongside young people with lived experience can help develop more engaging and accessible interventions that avoid young people trying to hide their parents’ substance use and control their situations by themselves.

### Strengths and Limitations

To our knowledge, this is the first comprehensive systematic review of children and young people’s coping strategies, perceived impacts, and experiences of parental alcohol and drug use. Our work drew on multiple qualitative studies, from a range of different countries, ethnicities, and ages. However, the data was not presented in a way to fully understand the complexity and issues of diversity among children and young people. Further research is needed on diversity in investigating the effects of parental substance use among children. The findings from this systematic review provide a broad understanding into the lives of those that have experienced parental substance use, which is important for practice and policy implications. Since we were limited to quotes that were included in the original studies, our study may not be fully inclusive of all perspectives. Included studies tended to report on the negative experiences and impacts, with only minor acknowledgement that not all children and young people experienced abuse and neglect. This is important to counter judgmental stereotyping and stigma towards parents who use substances, since not all parents who use substances become violent and abusive towards their children. Nevertheless, we found that the unpredictability in parental substance use and relationships can have emotional and social impacts on children and young people in the absence of abuse. More formal, ongoing, supportive, and child-centered interventions therefore need to be developed. See Table 3 for a further summary of the implications and recommendations for practice, policy, and research.

**Table 3.** Implications and Recommendations for Practice, Policy, and Research.

*Practitioners* are recommended to provide ongoing emotional and social support to young people alongside strategies to reduce parental substance use, as impacts can extend beyond periods of parental substance use. We encourage practitioners to be mindful of how stigma can exacerbate experiences for children and young people, including discriminatory behaviors within their own practice, especially to those who display externalized behaviors. Practitioners who adopt stigma-reducing and trauma-informed practices may find that the needs of children and young people are addressed. Relational practices, including building trusting relationships and allowing time and space for children and young people are seen as important. Training for all practitioners would enable them to deliver effective support for children and young people who experience parental substance use.
*Policies* should seek to address childhood socioeconomic conditions such as poverty to support the effectiveness of child-focused interventions. Polices which ensure that all local authorities provide specialist services for those impacted by parental substance use allow children and young people to have fairer access to support across the country.
*Researchers* are recommended to develop effective, feasible, and acceptable coproduced interventions that promote children and young people’s emotional and social resilience as well as reduce stigma. More research is also needed exploring diversity within this population.

Several limitations should be acknowledged in interpreting our findings. Firstly, we defined children and young people up until the age of 25, with several studies including older participants. This raises issues of retrospective accounts and recall bias, as well as viewing experiences through a young adult-lens that can alter how childhood experiences are interpreted (Gil-González et al., 2007). However, it has been argued that adolescence should cover the ages of 10 to 24 years as neurocognitive maturation continues past 20 years (Sawyer et al., 2018), as well as acknowledgement that there is a need for children’s services to go up to the age of 25 years to ease transitions into adult services (National Health Service, 2019). Additionally, most young people who were recruited into these studies were already known to services or had previously received support, therefore we did not capture the voices of those who had not had any support and it is likely that the views of those who experience non-dependent parental substance use was also missed. While no studies were excluded based on quality, over a third of the studies were rated as satisfactory on quality or relevance to the review aims, so focus was based initially on the key papers, with satisfactory papers supplementing the synthesis.

## Conclusion

The synthesis findings emphasize that children and young people who experience parental substance use are trying to manage and mitigate vulnerabilities and be resilient to unpredictable, adverse, and often stigmatizing experiences. While it is not a child’s role to have to resist and cope with the negative impacts of parental substance use, they are trying to do this anyway, often without formal support. Unfortunately, some of their strategies or externalized behaviors are inconsistent with societal norms and/or fail to produce the desired results. Therefore, alongside interventions to reduce parental risk, we need to work with children and young people to understand what strategies and resources will allow them to better cope with the social and emotional impacts of parental substance use. Additionally, we need to build resilient and non-stigmatizing systems surrounding the child through relational and trauma-informed practices.

## Supplemental Material

sj-docx-1-tva-10.1177_15248380221134297 – Supplemental material for A Systematic Review of Qualitative Studies Exploring Lived Experiences, Perceived Impact, and Coping Strategies of Children and Young People Whose Parents Use Substances Click here for additional data file. Supplemental material, sj-docx-1-tva-10.1177_15248380221134297 for A Systematic Review of Qualitative Studies Exploring Lived Experiences, Perceived Impact, and Coping Strategies of Children and Young People Whose Parents Use Substances by Cassey Muir, Emma A. Adams, Vivienne Evans, Emma Geijer-Simpson, Eileen Kaner, Sophie M. Phillips, Domna Salonen, Deborah Smart, Lizzy Winstone and Ruth McGovern in Trauma, Violence, & Abuse

## References

[bibr1-15248380221134297] Adamson J. Templeton L. (2012). Silent voices: Supporting children and young people affected by parental alcohol misuse. The Office of the Children’s Commissioner.

[bibr2-15248380221134297] Adjei N. K. Schlüter D. K. Straatmann V. S. Melis G. Fleming K. M. McGovern R. Howard L. M. Kaner E. Wolfe I. Taylor-Robinson D. C. (2022). Impact of poverty and family adversity on adolescent health: A multi-trajectory analysis using the UK Millennium Cohort Study. The Lancet Regional Health – Europe, 13, 100279. 10.1016/j.lanepe.2021.100279 35199082 PMC8841277

[bibr3-15248380221134297] Ahuja A. Orford J. Copello A. (2003). Understanding how families cope with alcohol problems in the UK West Midlands Sikh community. Contemporary Drug Problems, 30(4), 839–873. 10.1177/009145090303000406

[bibr4-15248380221134297] Alexanderson K. Näsman E. (2017). Children’s experiences of the role of the other parent when one parent has addiction problems. Drugs: Education, Prevention and Policy, 24(1), 32–39. 10.1080/09687637.2016.1218824

[bibr5-15248380221134297] Arai L. Shaw A. Feder G. Howarth E. MacMillan H. Moore T. H. M. Stanley N. Gregory A. (2021). Hope, agency, and the lived experience of violence: A qualitative systematic review of children’s perspectives on domestic violence and abuse. Trauma, Violence, & Abuse, 22(3), 427–438. 10.1177/1524838019849582 PMC8165749 31262231

[bibr6-15248380221134297] Backett-Milburn K. Wilson S. Bancroft A. Cunningham-Burley S. (2008). Challenging childhoods: Young people’s accounts of ‘getting by’ in families with substance use problems. Childhood, 15(4), 461–479. 10.1177/0907568208097202

[bibr7-15248380221134297] Bancroft A. Wilson S. (2007). The ‘risk gradient’ in policy on children of drug and alcohol users: Framing young people as risky. Health, Risk and Society, 9(3), 311–322. 10.1080/13698570701488837

[bibr8-15248380221134297] Bancroft A. Wilson S. Cunningham-Burley S. Backett-Milburn K. Masters H. (2004). Parental drug and alcohol misuse: Resilience and transition among young people. Retrieved July 11, 2019, from https://www.jrf.org.uk/sites/default/files/jrf/migrated/files/1859352499.pdf

[bibr9-15248380221134297] Barnard M. Barlow J. (2003). Discovering parental drug dependence: Silence and disclosure. Children and Society, 17(1), 45–56. 10.1002/chi.727

[bibr10-15248380221134297] Bernays S. Houmøller K. (2011). See me, not just the problem: Hiding, telling and coping with a difficult family life. London School of Hygiene and Tropical Medicine.

[bibr11-15248380221134297] Bickelhaupt S. E. Lohman B. J. Neppl T. K. (2021). The influence of parental alcoholism on parent–adolescent relationships from adolescence into emerging adulthood: A qualitative inquiry. Emerging Adulthood, 9(2), 117–131. 10.1177/2167696818824186

[bibr12-15248380221134297] Boyd H. McKernon S. Mullin B. Old A. (2012). Improving healthcare through the use of co-design. The New Zealand Medical Journal, 125(1357), 76–87. 22854362

[bibr13-15248380221134297] Britten N. Pope C. (2012). Medicine taking for asthma: A worked example of meta-ethnography. In Hannes K. Lockwood C. (Eds.), Synthesizing qualitative research: Choosing the right approach (pp. 41–57). Wiley-Blackwell.

[bibr14-15248380221134297] Cameron D. (2011). Troubled families speech. Retrieved December 9, 2021, from https://www.gov.uk/government/speeches/troubled-families-speech

[bibr15-15248380221134297] Canfield M. Radcliffe P. Marlow S. Boreham M. Gilchrist G. (2017). Maternal substance use and child protection: A rapid evidence assessment of factors associated with loss of child care. Child Abuse & Neglect, 70, 11–27. 10.1016/j.chiabu.2017.05.005 28551458

[bibr16-15248380221134297] Cargo M. Mercer S. L. (2008). The value and challenges of participatory research: Strengthening its practice. Annual Review of Public Health, 29, 325–350. 10.1146/annurev.publhealth.29.091307.083824 18173388

[bibr17-15248380221134297] Children’s Commissioner’s Office. (2020). Childhood Vulnerability in England. Retrieved January 10, 2022, from https://www.childrenscommissioner.gov.uk/chldrn/

[bibr18-15248380221134297] Christensen E. (1997). Aspects of a preventive approach to support children of alcoholics. Child Abuse Review, 6(1), 24–34. 10.1002/(SICI)1099-0852(199703)6:1<24::AID-CAR279>3.0.CO;2-Z

[bibr19-15248380221134297] Critical Appraisal Skills Programme. (2018). CASP qualitative studies checklist. Retrieved November 13, 2019, from https://casp-uk.b-cdn.net/wp-content/uploads/2018/03/CASP-Qualitative-Checklist-2018_fillable_form.pdf

[bibr20-15248380221134297] Davies E. L. Martin J. Foxcroft D. R. (2015). Development and acceptability of a co-produced online intervention to prevent alcohol misuse in adolescents: A think aloud study. JMIR human Factors, 2(2), e13. https://humanfactors.jmir.org/2015/2/e13/ (accession no. 27025403). 10.2196/humanfactors.4452 PMC4797700 27025403

[bibr21-15248380221134297] D’Costa M. Lavalekar A. (2021). A qualitative inquiry into the coping strategies of Goan adolescents living with alcoholic parents. Journal of Indian Association for Child and Adolescent Mental Health, 17(3), 12–36.

[bibr22-15248380221134297] DeJean D. Giacomini M. Simeonov D. Smith A. (2016). Finding qualitative research evidence for health technology assessment. Qualitative Health Research, 26(10), 1307–1317. 10.1177/1049732316644429 27117960

[bibr23-15248380221134297] Díaz R. Gual A. García M. Arnau J. Pascual F. Cañuelo B. Rubio G. Dios Y. Fernández-Eire M. C. Valdés R. Garbayo I. (2008). Children of alcoholics in Spain: From risk to pathology. Social Psychiatry and Psychiatric Epidemiology, 43(1), 1–10. 10.1007/s00127-007-0264-2 17932609

[bibr24-15248380221134297] Dixon-Woods M. Sutton A. Shaw R. Miller T. Smith J. Young B. Bonas S. Booth A. Jones D. (2007). Appraising qualitative research for inclusion in systematic reviews: A quantitative and qualitative comparison of three methods. Journal of Health Services Research and Policy, 12(1), 42–47. 10.1258/135581907779497486 17244397

[bibr25-15248380221134297] Dundas I. (2000). Cognitive/affective distancing as a coping strategy of children of parents with a drinking problem. Alcoholism Treatment Quarterly, 18(4), 85–98. 10.1300/J020v18n04_07

[bibr26-15248380221134297] European Monitoring Centre for Drugs and Drug Addiction. (2008). Drugs and vulnerable groups of young people, Selected issue. Retrieved January 10, 2022, from http://www.emcdda.europa.eu/publications/selected-issues/vulnerable-young

[bibr27-15248380221134297] Felitti V. J. Anda R. F. Nordenberg D. Williamson D. F. Spitz A. M. Edwards V. Koss M. P. Marks J. S. (1998). Relationship of childhood abuse and household dysfunction to many of the leading causes of death in adults. The Adverse Childhood Experiences (ACE) Study. American Journal of Preventive Medicine, 14(4), 245–258. 10.1016/s0749-3797(98)00017-8 9635069

[bibr28-15248380221134297] Fellin L. C. Callaghan J. E. Alexander J. H. Harrison-Breed C. Mavrou S. Papathanasiou M. (2019). Empowering young people who experienced domestic violence and abuse: The development of a group therapy intervention. Clinical Child Psychology and Psychiatry, 24(1), 170–189. 10.1177/1359104518794783 30156129

[bibr29-15248380221134297] Ferguson H. Warwick L. Disney T. Leigh J. Cooner T. S. Beddoe L. (2022). Relationship-based practice and the creation of therapeutic change in long-term work: Social work as a holding relationship. Social Work Education, 41(2), 209–227. 10.1080/02615479.2020.1837105

[bibr30-15248380221134297] Fraser C. McIntyre A. Manby M. (2009). Exploring the impact of parental drug/alcohol problems on children and parents in a midlands county in 2005/06. British Journal of Social Work, 39(5), 846–866. 10.1093/bjsw/bcn016

[bibr31-15248380221134297] Galligan K. Comiskey C. M. (2019). Hidden harms and the number of children whose parents misuse substances: A stepwise methodological framework for estimating prevalence. Substance Use and Misuse, 54(9), 1429–1437. 10.1080/10826084.2019.1584224 30942121

[bibr32-15248380221134297] Gil-González D. Vives-Cases C. Ruiz M. T. Carrasco-Portiño M. Álvarez-Dardet C. (2007). Childhood experiences of violence in perpetrators as a risk factor of intimate partner violence: A systematic review. Journal of Public Health, 30(1), 14–22. 10.1093/pubmed/fdm071 17986717

[bibr33-15248380221134297] Goddard A. (2021). Adverse childhood experiences and trauma-informed care. Journal of Pediatric Health Care, 35(2), 145-155. 10.1016/j.pedhc.2020.09.001 33129624

[bibr34-15248380221134297] Goldson B. Muncie J. (2015). Youth Crime and Justice. London, UK: Sage.

[bibr35-15248380221134297] Gorin S. (2004). Understanding what children say about living with domestic violence, parental substance misuse or parental health problems. Joseph Rowntree Foundation.

[bibr36-15248380221134297] Hagström A. S. Forinder U. (2019). ‘If I whistled in her ear she’d wake up’: Children’s narration about their experiences of growing up in alcoholic families. Journal of Family Studies. 10.1080/13229400.2019.1699849

[bibr37-15248380221134297] Hill L. (2015). ‘Don't make us talk!’: Listening to and learning from children and young people living with parental alcohol problems. Children & Society, 29(5), 344-354. 10.1111/chso.12064

[bibr38-15248380221134297] Hill M. Laybourn A. Brown J. (1996). Children whose parents misuse alcohol: A study of services and needs. Child and Family Social Work, 1, 159-167. 10.1111/j.1365-2206.1996.tb00022.x

[bibr39-15248380221134297] Hogan D. Higgins L. (2001). When parents use drugs: Key findings from a study of children in the area of drug-using parents. Children’s Research Centre.

[bibr40-15248380221134297] Holmila M. J. Itäpuisto M. Ilva M. (2011). Invisible victims or competent agents: Opinions and ways of coping among children aged 12-18 years with problem drinking parents. Drugs: Education, Prevention and Policy, 18(3), 179-186. 10.3109/09687637.2010.493168

[bibr41-15248380221134297] Hussong A. Chassin L. (2004). Stress and coping among children of alcoholic parents through the young adult transition. Development and Psychopathology, 16(4), 985-1006. 10.1017/S0954579404040106 15704824 PMC3159037

[bibr42-15248380221134297] Houmøller K. Bernays S. Wilson S. Rhodes T. (2011). Juggling harms: Coping with parental substance misuse. London School of Hygeine & Tropical Medicine.

[bibr43-15248380221134297] Institute of Alcohol Studies, Adfam, & Alcohol Focus Scotland. (2017). “Like sugar for adults”: The effect of non-dependent parental drinking on children & families. Institute of Alcohol Studies, Adfam & Alcohol Focus Scotland.

[bibr44-15248380221134297] Johnson S. D. (2013). African American adolescents' interactions with their substance-using mothers. Families in Society, 94(2), 121-128. 10.1606/1044-3894.4288

[bibr45-15248380221134297] Kotova A. (2020). Beyond courtesy stigma: Towards a multi-faceted and cumulative model of stigmatisation of families of people in prison. Forensic Science International: Mind and Law, 1, 100021. 10.1016/j.fsiml.2020.100021

[bibr46-15248380221134297] Kroll B. (2004). Living with an elephant: Growing up with parental substance misuse. Child & Family Social Work, 9(2), 129-140. 10.1111/j.1365-2206.2004.00325.x

[bibr47-15248380221134297] Muir C. McGovern R. Kaner E. (2022). Stigma and young people whose parents use substances. In Addison M. McGovern W. McGovern R. (Eds.), Drugs, identity and stigma. London, UK: Palgrave Macmillan. 10.1007/978-3-030-98286-7_8

[bibr48-15248380221134297] Muir C. McGovern R. Kaner E. Geijer-Simpson E. Smart D. Kidger J. Winstone L. Evans V. Phillips S. M. Salonen D. Adams E. (2019). A systematic review of qualitative studies exploring lived experiences, impacts and coping strategies of children and young people affected by parental substance misuse. PROSPERO. Retrieved June 11, 2019, from https://www.crd.york.ac.uk/prospero/display_record.php?ID=CRD42019137486 10.1177/15248380221134297 PMC10594843 36384375

[bibr49-15248380221134297] Lead Author’s Name. (2022). Stigma and young people whose parents use substances. In Addison M. McGovern W. McGovern R. (Eds.), Drugs, Identity and Stigma. London, UK: Palgrave Macmillan.

[bibr50-15248380221134297] Lewis Q. J. Smith B. D. Offiong A. Prioleau M. Powell T. W. (2021). When a house is never a home: Housing instability among youth affected by parental drug abuse. Child Abuse & Neglect, 118, 105131. 10.1016/j.chiabu.2021.105131 34118586 PMC8341207

[bibr51-15248380221134297] Livingston J. D. Milne T. Fang M. L. Amari E. (2012). The effectiveness of interventions for reducing stigma related to substance use disorders: A systematic review. Addiction, 107(1), 39–50. 10.1111/j.1360-0443.2011.03601.x PMC3272222 21815959

[bibr52-15248380221134297] Malpass A. Shaw A. Sharp D. Walter F. Feder G. Ridd M. Kessler D. (2009). “Medication career” or “moral career”? The two sides of managing antidepressants: A meta-ethnography of patients' experience of antidepressants. Social Science and Medicine, 68(1), 154–168. 10.1016/j.socscimed.2008.09.068 19013702

[bibr53-15248380221134297] Marmot M. Allen J. Boyce T. Goldblatt P. Morrison J. (2020). Health equity in England: The Marmot review 10 years on. Retrieved January 10, 2022, from https://www.health.org.uk/publications/reports/the-marmot-review-10-years-on

[bibr54-15248380221134297] McGovern R. Gilvarry E. Addison M. Alderson H. Carr L. Geijer-Simpson E. Hrisos N. Lingham R. Minos D. Smart D. Kaner E. (2018). Addressing the impact of nondependent parental substance misuse upon children: A rapid review of the evidence of prevalence impact and effective interventions. Public Health England.

[bibr55-15248380221134297] McGovern R. Newham J. J. Addison M. T. Hickman M. Kaner E. F. S. (2021). Effectiveness of psychosocial interventions for reducing parental substance misuse. Cochrane Database of Systematic Reviews, 2021(3) 1–104. 10.1002/14651858.CD012823.pub2 PMC8094759 33723860

[bibr56-15248380221134297] McGovern R. Smart D. Alderson H. Araújo-Soares V. Brown J. Buykx P. Evans V. Fleming K. Hickman M. Macleod J. Meier P. Kaner E. (2021). Psychosocial interventions to improve psychological, social and physical wellbeing in family members affected by an adult relative’s substance use: A systematic search and review of the evidence. International Journal of Environmental Research and Public Health, 18(4), 1793. 10.3390/ijerph18041793 33673199 PMC7918716

[bibr57-15248380221134297] McGuire M. (2002). Keeping it quiet: Children and families in Greater Govan affected by parental drug use. Aberlour Child Care Trust.

[bibr58-15248380221134297] McLaughlin A. O'Neill T. McCartan C. Percy A. McCann M. Perra O. Higgins K. (2015). Parental alcohol use and resilience in young people in Northern Ireland: A study of family, peer and school processes. Institute of Child Care Research.

[bibr59-15248380221134297] Molina B. S. G. Donovan J. E. Belendiuk K. A. (2010). Familial loading for alcoholism and offspring behavior: Mediating and moderating influences. Alcoholism: Clinical and Experimental Research, 34(11), 1972–1984. 10.1111/j.1530-0277.2010.01287.x 20659068 PMC2965318

[bibr60-15248380221134297] Moore T. McArthur M. Noble-Carr D. (2011). Different but the same? Exploring the experiences of young people caring for a parent with an alcohol or other drug issue. Journal of Youth Studies, 14(2), 161–177. 10.1080/13676261.2010.522561

[bibr61-15248380221134297] Moore T. Noble-Carr D. McArthur M. (2010). Who cares? Young people with parents who use alcohol or other drugs talk about their experiences with services. Family Matters, 85(1), 18–27.

[bibr62-15248380221134297] Mudau T. J. (2018). Challenges faced by young people living with alcoholic parents: A case of Tzaneen around Mamokgadi. African Renaissance, 15(4), 253–271. https://hdl.handle.net/10520/EJC-137568f812

[bibr63-15248380221134297] Munford R. (2022). Children and young people in the care system: Relational practice in working with transitions and challenges. Australian Social Work, 75(1), 1–4. 10.1080/0312407X.2021.1989003

[bibr64-15248380221134297] Murray B. L. (1998). Perceptions of adolescents living with parental alcoholism. Journal of Psychiatric and Mental Health Nursing, 5(6), 525–534. 10.1046/j.1365-2850.1998.560525.x 10076283

[bibr65-15248380221134297] National Health Service. (2019). The NHS long term plan. Retrieved January 10, 2022, from https://www.longtermplan.nhs.uk/publication/nhs-long-term-plan/

[bibr66-15248380221134297] Nattala P. Murthy P. Weiss M. G. Leung K. S. Christopher R. Jessy Sharoon V. Sumegha S. (2020). Experiences and reactions of adolescent offspring to their fathers’ heavy drinking: A qualitative study from an urban metropolis in India. Journal of Ethnicity in Substance Abuse, 21, 284–303. 10.1080/15332640.2020.1747041 32324108

[bibr67-15248380221134297] O’Connor L. Forrester D. Holland S. Williams A. (2014). Perspectives on children’s experiences in families with parental substance misuse and child protection interventions. Children and Youth Services Review, 38, 66–74. 10.1016/j.childyouth.2014.01.008

[bibr68-15248380221134297] Offiong A. Powell T. W. Lewis Q. Smith B. Prioleau M. (2020). “I missed open arms”: The need for connectedness among Black youth affected by parental drug use. Children and Youth Services Review, 114, 1–7. 10.1016/j.childyouth.2020.105072 PMC7326313 32606485

[bibr69-15248380221134297] Park S. Schepp K. G. (2015). A systematic review of research on children of alcoholics: Their inherent resilience and vulnerability. Journal of Child and Family Studies, 24, 1222–1231. 10.1007/s10826-014-9930-7

[bibr70-15248380221134297] Park S. Schepp K. G. (2017). The patterns of adaptation while growing up under parental alcoholism: A grounded theory. Journal of Child and Family Studies, 26(7), 1875–1887. 10.1007/s10826-017-0717-5

[bibr71-15248380221134297] Park S. Schepp K. G. (2018). A theoretical model of resilience capacity: Drawn from the words of adult children of alcoholics. Nursing Forum, 53(3), 314–323. 10.1111/nuf.12255 29691871

[bibr72-15248380221134297] Park S. Schepp K. G. Park D. (2016). Living with appending a scarlet letter: The lifelong suffering of children of alcoholics in South Korea. Journal of Ethnicity in Substance Abuse, 15(4), 367–385. 10.1080/15332640.2016.1175989 27230610

[bibr73-15248380221134297] Pisinger V.S.C. Tolstrup J.S. (2022). Are emotional symptoms and depression among young people with parental alcohol problems modified by socioeconomic position? European Child & Adolescent Psychiatry, 31, 747–755. 10.1007/s00787-020-01716-z 33432403

[bibr74-15248380221134297] Powell T. W. Willis K. Smith B. Lewis Q. Offiong A. (2021). “Don't close the door on them”: Recruiting and retaining vulnerable Black adolescents in prevention research. Journal of Community Psychology, 49(5), 994–1009. 10.1002/jcop.22584 33937999 PMC8222182

[bibr75-15248380221134297] QSR International Pty Ltd. (2018). NVivo (Version 12). Retrieved February 4, 2020 from https://www.qsrinternational.com/nvivo-qualitative-data-analysis-software/home

[bibr76-15248380221134297] Ramírez Dávila A. S. Naal A. R. Salinas E. K. Pérez C. A. (2014). The perception of a father's alcoholism through the eyes of his children. Health and Addictions/Salud y Drogas, 14(2), 109–120. 10.21134/haaj.v14i2.219

[bibr77-15248380221134297] Reupert A. Goodyear M. Maybery D. (2012). Engaging with, and understanding children whose parents have a dual diagnosis. Child and Adolescent Mental Health, 17(3), 153–160. 10.1111/j.1475-3588.2011.00622.x 32847265

[bibr78-15248380221134297] Ronel N. Haimoff-Ayali R. (2010). Risk and resilience: The family experience of adolescents with an addicted parent. International Journal of Offender Therapy and Comparative Criminology, 54(3), 448–472. 10.1177/0306624X09332314 19270268

[bibr79-15248380221134297] Ronel N. Levy-Cahana M. (2011). Growing-up with a substance-dependent parent: Development of subjective risk and protective factors. Substance Use & Misuse, 46(5), 608–619. 10.3109/10826084.2010.527417 20973694

[bibr80-15248380221134297] Sawyer S. M. Azzopardi P. S. Wickremarathne D. Patton G. C. (2018). The age of adolescence. The Lancet Child & Adolescent Health, 2(3), 223–228. 10.1016/S2352-4642(18)30022-1 30169257

[bibr81-15248380221134297] Shaw R. L. Booth A. Sutton A. J. Miller T. Smith J. A. Young B. Jones D. R. Dixon-Woods M. (2004). Finding qualitative research: An evaluation of search strategies. BMC Medical Research Methodology, 4(1), 5. 10.1186/1471-2288-4-5 15070427 PMC385230

[bibr82-15248380221134297] Silva S. Padilha M. (2013). Alcoholism in adolescents' life histories: An analysis in the light of social representations. Texto & Contexto – Enfermagem, 22, 576–584. 10.1590/S0104-07072013000300002

[bibr83-15248380221134297] Silva S. Padilha M. Araujo J. (2013). Interaction of the teen with the alcoholic relative and its influence for alcoholic addiction. Journal of Nursing UFPE On Line, 8(1), 59–68. 10.5205/reuol.4843-39594-1-SM.0801201409

[bibr84-15248380221134297] Tamutienė I. Jogaitė B. (2019). Disclosure of alcohol-related harm: Children’s experiences. NAD Nordic Studies on Alcohol and Drugs, 36(3), 209–222. 10.1177/1455072518807789 PMC7434161 32934561

[bibr85-15248380221134297] Templeton L. Velleman R. Hardy E. Boon S. (2009). Young people living with parental alcohol misuse and parental violence: ‘No-one has ever asked me how I feel in any of this’. Journal of Substance Use, 14(3–4), 139–150. 10.1080/14659890802624287

[bibr86-15248380221134297] Templeton L. Velleman R. Russell C. (2010). Psychological interventions with families of alcohol misusers: A systematic review. Addiction Research & Theory, 18, 616–648. 10.3109/16066350903499839

[bibr87-15248380221134297] Thomas J. Harden A. (2008). Methods for the thematic synthesis of qualitative research in systematic reviews. BMC Medical Research Methodology, 8(1), 45. 10.1186/1471-2288-8-45 18616818 PMC2478656

[bibr88-15248380221134297] Tinnfält A. Eriksson C. Brunnberg E. (2011). Adolescent children of alcoholics on disclosure, support, and assessment of trustworthy adults. Child and Adolescent Social Work Journal, 28(2), 133–151. 10.1007/s10560-011-0225-1

[bibr89-15248380221134297] Tinnfält A. Fröding K. Larsson M. Dalal K. (2018). “I feel it in my heart when my parents fight”: Experiences of 7–9-year-old children of alcoholics. Child and Adolescent Social Work Journal, 35(5), 531–540. 10.1007/s10560-018-0544-6 30220781 PMC6133169

[bibr90-15248380221134297] Turning Point. (2006). Bottling it up: The effects of alcohol misuse on children, parents and families. Turning Point.

[bibr91-15248380221134297] Velleman R. Templeton L. (2007). Understanding and modifying the impact of parents’ substance misuse on children. Advances in Psychiatric Treatment, 13, 79–89. 10.1192/apt.bp.106.002386

[bibr92-15248380221134297] Velleman R. Templeton L. (2016). Impact of parents’ substance misuse on children: An update. British Journal of Psych Advances, 22, 108–117.

[bibr93-15248380221134297] Velleman R. Templeton L. Reuber D. Klein M. Moesgen D. (2008). Domestic abuse experienced by young people living in families with alcohol problems: Results from a cross-European study. Child Abuse Review, 17(6), 387–409. 10.1002/car.1047

[bibr94-15248380221134297] Wangensteen T. Bramness J. G. Halsa A. (2019). Growing up with parental substance use disorder: The struggle with complex emotions, regulation of contact, and lack of professional support. Child and Family Social Work, 24(2), 201–208. 10.1111/cfs.12603

[bibr95-15248380221134297] Wangensteen T. Halsa A. Bramness J. G. (2020). Creating meaning to substance use problems: A qualitative study with patients in treatment and their children. Journal of Substance Use, 25(4), 382–386. 10.1080/14659891.2020.1715497

[bibr96-15248380221134297] Wangensteen T. Westby L. C. L. (2019). Breaking the cycle: Young people’s stories of protection and support while growing up with parental substance use disorder. Child Care in Practice, 27(2), 155–168. 10.1080/13575279.2019.1664989

[bibr97-15248380221134297] Werner E. E. Johnson J. L. (2004). The role of caring adults in the lives of children of alcoholics. Substance Use & Misuse, 39(5), 699–720. 10.1081/JA-120034012 15202805

[bibr98-15248380221134297] Wilson S. Cunningham-Burley S. Bancroft A. Backett-Milburn K. (2008). 'Joined up' thinking? Unsupported 'fast-track' transitions in the context of parental substance use. Journal of Youth Studies, 11(3), 283–299. 10.1080/13676260801946423

[bibr99-15248380221134297] Wilson S. Cunningham-Burley S. Bancroft A. Backett-Milburn K. (2012). The consequences of love: Young people and family practices in difficult circumstances. Sociological Review, 60(1), 110–128. 10.1111/j.1467-954X.2011.02049.x

[bibr100-15248380221134297] Wlodarczyk O. Schwarze M. Rumpf H. Metzner F. Pawils S. (2017). Protective mental health factors in children of parents with alcohol and drug use disorders: A systematic review. PLoS ONE, 12(6), e0179140. 10.1371/journal.pone.0179140 PMC5469455 28609440

[bibr101-15248380221134297] Yusay C. T. C. Canoy N. A. (2019). Healing the hurt amid the drug war: Narratives of young urban poor Filipinos in recovering families with parental drug use. International Journal of Drug Policy, 68, 124–131. 10.1016/j.drugpo.2018.10.009 30551812

