# Supplementary material for: A Systematic Review of Qualitative Studies Exploring Lived Experiences, Perceived Impact, and Coping Strategies of Children and Young People Whose Parents Use Substances
Source: Trauma Violence Abuse. 2022 Nov 17;24(5):3629–46. doi: 10.1177/15248380221134297 (PMC10594843; doi:10.1177/15248380221134297)
Supplement: sj-docx-1-tva-10.1177_15248380221134297 – Supplemental material for A Systematic Review of Qualitative Studies Exploring Lived Experiences, Perceived Impact, and Coping Strategies of Children and Young People Whose Parents Use Substances [file sj-docx-1-tva-10.1177_15248380221134297.docx]

**Supplemental Materials**

**Supplemental Appendix A. Search Strategy**

**ProQuest (International Bibliography of the Social Sciences, Social Science Database, Sociology Collection: Sociology Database)**

*Children and Young People*

(ab(("young person" OR "young people" OR young* OR child* OR youth OR adolescen* OR teen* OR pre?teen OR "young adult*" OR offspring OR juvenile* OR pubescen* OR "school child*" OR student OR boy* OR girl* OR pupil)) OR (MAINSUBJECT.EXACT("Young adults") OR MAINSUBJECT.EXACT("Children of alcoholics") OR MAINSUBJECT.EXACT("Teenagers") OR MAINSUBJECT.EXACT("Children & youth"))) AND PEER(yes)

*Parental Substance Use*

(NOFT((("alcohol consumption" OR "alcohol misuse" OR "alcohol use*" OR "misuse alcohol" OR "alcohol intoxicat*" OR "alcohol drinking" OR "alcohol disorder*" OR "binge drinking" OR "social drinking" OR "risky drinking" OR "substance misuse" OR "substance use*" OR "misuse substances" OR "substance disorder" OR "substance abuse" OR "hazardous drinking" OR "hazardous alcohol" OR "harmful alcohol" OR "harmful drinking" OR "alcohol dependen*" OR "dependent drinking" OR alcoholic OR alcoholism OR "drug consumption" OR "drug misuse" OR "drug use*" OR "misuse drugs" OR "drug disorder*" OR "drug dependen*" OR "illicit drugs" OR "alcohol or other drug*") NEAR/2 (parent* OR father* OR paternal OR step?father OR mother* OR maternal OR step?mother OR carer* OR care?giver OR foster?parent* OR grand?parent* OR grand?mother* OR grand?father* OR family OR families))) OR (MAINSUBJECT.EXACT("Children of alcoholics"))) AND PEER(yes)

*Qualitative Research*

(ab((interview* OR theme* OR "thematic analysis" OR qualitative OR "nursing research methodology" OR questionnaire OR ethnograph* OR ethnonursing OR "ethnological research" OR phenomenol* OR "grounded theor*" OR "grounded stud*" OR "grounded research" OR "grounded analys?s" OR "Life Stor*" OR "Women's Stor*" OR emic OR etic OR hermeneutic OR heuristic OR semiotic OR "data saturat*" OR "participant observ*" OR "social construct*" OR Postmodern* OR "Post structural*" OR feminis* OR interpret* OR "action research" OR "co-operative inquir*" OR Humanistic OR Existential OR Experiential OR Paradigm* OR "field stud*" OR "field research" OR "human science" OR "biographical method" OR "theoretical sampl*" OR "Purposive sampl*" OR "open-ended account*" OR "unstructured account" OR narrative* OR text* OR "life world" OR "conversation analys?s" OR "theoretical saturation" OR "lived experience" OR "life experience" OR "living with" OR "cluster sampl*" OR "observational method*" OR "content analysis" OR "constant comparative" OR "discourse analys?s" OR "discurs* analys?s" OR "narrative analys?s" OR Heidegger* OR colaizzi* OR spiegelberg* OR "Van manen*" OR "Van Kaam*" OR "merleau ponty*" OR Husserl* OR Foucault* OR corbin* OR strauss* OR glaser*)) OR (MAINSUBJECT.EXACT("Qualitative research") OR MAINSUBJECT.EXACT("Phenomenology") OR MAINSUBJECT.EXACT("Grounded theory") OR MAINSUBJECT.EXACT("Interviews") OR MAINSUBJECT.EXACT("Personal experiences") OR MAINSUBJECT.EXACT("Ethnography") OR MAINSUBJECT.EXACT("Focus groups") OR MAINSUBJECT.EXACT("Discourse analysis"))) AND PEER(yes)

Above combined with AND.

Found 3/5 papers in database: does not find Bancroft et al. (2004) and Barnard & Barlow (2003) due to no abstract or thesaurus terms.

**ProQuest (Sociology Collection: Sociological Abstracts)**

*Children and Young People*

(ab(("young person" OR "young people" OR young* OR child* OR youth OR adolescen* OR teen* OR pre?teen OR "young adult*" OR offspring OR juvenile* OR pubescen* OR "school child*" OR student OR boy* OR girl* OR pupil)) OR (MAINSUBJECT.EXACT.EXPLODE("Children") OR MAINSUBJECT.EXACT("Adolescents") OR MAINSUBJECT.EXACT("Young Adults"))) AND PEER(yes)

*Parental Substance Use*

(NOFT((("alcohol consumption" OR "alcohol misuse" OR "alcohol use*" OR "misuse alcohol" OR "alcohol intoxicat*" OR "alcohol drinking" OR "alcohol disorder*" OR "binge drinking" OR "social drinking" OR "risky drinking" OR "substance misuse" OR "substance use*" OR "misuse substances" OR "substance disorder" OR "substance abuse" OR "hazardous drinking" OR "hazardous alcohol" OR "harmful alcohol" OR "harmful drinking" OR "alcohol dependen*" OR "dependent drinking" OR alcoholic OR alcoholism OR "drug consumption" OR "drug misuse" OR "drug use*" OR "misuse drugs" OR "drug disorder*" OR "drug dependen*" OR "illicit drugs" OR "alcohol or other drug*") NEAR/2 (parent* OR father* OR paternal OR step?father OR mother* OR maternal OR step?mother OR carer* OR care?giver OR foster?parent* OR grand?parent* OR grand?mother* OR grand?father* OR family OR families))) OR (MAINSUBJECT.EXACT("Drug Addiction") OR MAINSUBJECT.EXACT("Alcoholism"))) AND PEER(yes)

*Qualitative Research*

(ab((interview* OR theme* OR "thematic analysis" OR qualitative OR "nursing research methodology" OR questionnaire OR ethnograph* OR ethnonursing OR "ethnological research" OR phenomenol* OR "grounded theor*" OR "grounded stud*" OR "grounded research" OR "grounded analys?s" OR "Life Stor*" OR "Women's Stor*" OR emic OR etic OR hermeneutic OR heuristic OR semiotic OR "data saturat*" OR "participant observ*" OR "social construct*" OR Postmodern* OR "Post structural*" OR feminis* OR interpret* OR "action research" OR "co-operative inquir*" OR Humanistic OR Existential OR Experiential OR Paradigm* OR "field stud*" OR "field research" OR "human science" OR "biographical method" OR "theoretical sampl*" OR "Purposive sampl*" OR "open-ended account*" OR "unstructured account" OR narrative* OR text* OR "life world" OR "conversation analys?s" OR "theoretical saturation" OR "lived experience" OR "life experience" OR "living with" OR "cluster sampl*" OR "observational method*" OR "content analysis" OR "constant comparative" OR "discourse analys?s" OR "discurs* analys?s" OR "narrative analys?s" OR Heidegger* OR colaizzi* OR spiegelberg* OR "Van manen*" OR "Van Kaam*" OR "merleau ponty*" OR Husserl* OR Foucault* OR corbin* OR strauss* OR glaser*)) OR (MAINSUBJECT.EXACT("Group Research") OR MAINSUBJECT.EXACT("Phenomenology") OR MAINSUBJECT.EXACT("Grounded Theory") OR MAINSUBJECT.EXACT("Discourse Analysis") OR MAINSUBJECT.EXACT("Qualitative Methods") OR MAINSUBJECT.EXACT("Interviews") OR MAINSUBJECT.EXACT("Social Policy"))) AND PEER(yes)

Above combined with AND.

Found 5/5 papers in database.

**ProQuest (Sociology Collection: Applied Social Sciences Index and Abstracts)**

*Children and Young People*

(ab(("young person" OR "young people" OR young* OR child* OR youth OR adolescen* OR teen* OR pre?teen OR "young adult*" OR offspring OR juvenile* OR pubescen* OR "school child*" OR student OR boy* OR girl* OR pupil)) OR (MAINSUBJECT.EXACT("Adult children") OR MAINSUBJECT.EXACT("Adolescents") OR MAINSUBJECT.EXACT("Young adults") OR MAINSUBJECT.EXACT("Young people") OR MAINSUBJECT.EXACT("Children"))) AND PEER(yes)

*Parental Substance Use*

(NOFT((("alcohol consumption" OR "alcohol misuse" OR "alcohol use*" OR "misuse alcohol" OR "alcohol intoxicat*" OR "alcohol drinking" OR "alcohol disorder*" OR "binge drinking" OR "social drinking" OR "risky drinking" OR "substance misuse" OR "substance use*" OR "misuse substances" OR "substance disorder" OR "substance abuse" OR "hazardous drinking" OR "hazardous alcohol" OR "harmful alcohol" OR "harmful drinking" OR "alcohol dependen*" OR "dependent drinking" OR alcoholic OR alcoholism OR "drug consumption" OR "drug misuse" OR "drug use*" OR "misuse drugs" OR "drug disorder*" OR "drug dependen*" OR "illicit drugs" or "alcohol or other drug*") NEAR/2 (parent* OR father* OR paternal OR step?father OR mother* OR maternal OR step?mother OR carer* OR care?giver OR foster?parent* OR grand?parent* OR grand?mother* OR grand?father* OR family OR families))) OR (MAINSUBJECT.EXACT.EXPLODE("Problem drinkers") OR MAINSUBJECT.EXACT.EXPLODE("Abusers") OR MAINSUBJECT.EXACT.EXPLODE("Drug addicts"))) AND PEER(yes)

*Qualitative Research*

(ab((interview* OR theme* OR "thematic analysis" OR qualitative OR "nursing research methodology" OR questionnaire OR ethnograph* OR ethnonursing OR "ethnological research" OR phenomenol* OR "grounded theor*" OR "grounded stud*" OR "grounded research" OR "grounded analys?s" OR "Life Stor*" OR "Women's Stor*" OR emic OR etic OR hermeneutic OR heuristic OR semiotic OR "data saturat*" OR "participant observ*" OR "social construct*" OR Postmodern* OR "Post structural*" OR feminis* OR interpret* OR "action research" OR "co-operative inquir*" OR Humanistic OR Existential OR Experiential OR Paradigm* OR "field stud*" OR "field research" OR "human science" OR "biographical method" OR "theoretical sampl*" OR "Purposive sampl*" OR "open-ended account*" OR "unstructured account" OR narrative* OR text* OR "life world" OR "conversation analys?s" OR "theoretical saturation" OR "lived experience" OR "life experience" OR "living with" OR "cluster sampl*" OR "observational method*" OR "content analysis" OR "constant comparative" OR "discourse analys?s" OR "discurs* analys?s" OR "narrative analys?s" OR Heidegger* OR colaizzi* OR spiegelberg* OR "Van manen*" OR "Van Kaam*" OR "merleau ponty*" OR Husserl* OR Foucault* OR corbin* OR strauss* OR glaser*)) AND PEER(yes)) OR ((MAINSUBJECT.EXACT("Qualitative data") OR MAINSUBJECT.EXACT("Qualitative methods") OR MAINSUBJECT.EXACT("Emotional experiences") OR MAINSUBJECT.EXACT("Life experiences") OR MAINSUBJECT.EXACT("Personal experiences") OR MAINSUBJECT.EXACT("Qualitative analysis") OR MAINSUBJECT.EXACT("Focus groups") OR MAINSUBJECT.EXACT("Discourse analysis") OR MAINSUBJECT.EXACT("Focus group interviews") OR MAINSUBJECT.EXACT("Group interviewing") OR MAINSUBJECT.EXACT("Grounded theory") OR MAINSUBJECT.EXACT("Qualitative research") OR MAINSUBJECT.EXACT.EXPLODE("Phenomenology") OR MAINSUBJECT.EXACT.EXPLODE("Action research") OR MAINSUBJECT.EXACT("Structured interviews") OR MAINSUBJECT.EXACT("Semistructured interviews")) AND PEER(yes))

Above combined with AND.

Found 4/5 papers: does not find Bancroft et al. (2004) due to no abstract.

**OVID (Medline)**

*Children and Young People*

("young person" or "young people" or young* or child* or youth or adolescen* or teen* or pre?teen or "young adult*" or offspring or juvenile* or pubescen* or "school child*" or student or boy* or girl* or pupil).mp.

adolescent/ or young adult/ or exp child/

*Parental Substance Use*

(("alcohol consumption" or "alcohol misuse" or "alcohol use*" or "misuse alcohol" or "alcohol intoxicat*" or "alcohol drinking" or "alcohol disorder*" or "binge drinking" or "social drinking" or "risky drinking" or "substance misuse" or "substance use*" or "misuse substances" or "substance disorder" or "substance abuse" or "hazardous drinking" or "hazardous alcohol" or "harmful alcohol" or "harmful drinking" or "alcohol dependen*" or "dependent drinking" or alcoholic or alcoholism or "drug consumption" or "drug misuse" or "drug use*" or "misuse drugs" or "drug disorder*" or "drug dependen*" or "illicit drugs" or "alcohol or other drug*") adj2 (parent* or father* or paternal or step?father or mother* or maternal or step?mother or carer* or care?giver or foster?parent* or grand?parent* or grand?mother* or grand?father* or family or families)).mp.

alcoholics/ or "child of impaired parents"/ or drug users/

*Qualitative Research*

1 (theme$ or thematic).mp.

2 qualitative.af.

3 questionnaire$.mp.

4 ethnological research.mp.

5 ethnograph$.mp.

6 ethnonursing.af.

7 phenomenol$.af.

8 (grounded adj (theor$ or study or studies or research or analys?s)).af.

9 (life stor$ or women* stor$).mp.

10 (emic or etic or hermeneutic$ or heuristic$ or semiotic$).af. or (data adj1 saturat$).tw. or participant observ$.tw.

11 (social construct$ or (postmodern$ or post-structural$) or (post structural$ or poststructural$) or post modern$ or post-modern$ or feminis$ or interpret$).mp.

12 (action research or cooperative inquir$ or co operative inquir$ or co-operative inquir$).mp.

13 (humanistic or existential or experiential or paradigm$).mp.

14 (field adj (study or studies or research)).tw.

15 human science.tw.

16 biographical method.tw.

17 theoretical sampl$.af.

18 ((purpos$ adj4 sampl$) or (focus adj group$)).af.

19 (account or accounts or unstructured or openended or open ended or text$ or narrative$).mp.

20 (life world or life-world or conversation analys?s or personal experience$ or theoretical saturation).mp.

21 ((lived or life) adj experience$).mp.

22 cluster sampl$.mp.

23 observational method$.af.

24 content analysis.af.

25 (constant adj (comparative or comparison)).af.

26 ((discourse$ or discurs$) adj3 analys?s).tw.

27 narrative analys?s.af.

28 heidegger$.tw.

29 colaizzi$.tw.

30 spiegelberg$.tw.

31 (van adj manen$).tw.

32 (van adj kaam$).tw.

33 (merleau adj ponty$).tw.

34 husserl$.tw.

35 foucault$.tw.

36 (corbin$ adj2 strauss$).tw.

37 glaser$.tw.

38 living with.mp.

focus groups/ or interviews as topic/ or attitude/ or grounded theory/ or exp qualitative research/ or exp biography/ or Nursing Methodology Research/

Above combined with AND.

Found 1/1 papers in database.

**OVID (PsycINFO)**

*Children and Young People*

("young person" or "young people" or young* or child* or youth or adolescen* or teen* or pre?teen or "young adult*" or offspring or juvenile* or pubescen* or "school child*" or student or boy* or girl* or pupil).mp.

**No thesaurus terms

*Parental Substance Use*

(("alcohol consumption" or "alcohol misuse" or "alcohol use*" or "misuse alcohol" or "alcohol intoxicat*" or "alcohol drinking" or "alcohol disorder*" or "binge drinking" or "social drinking" or "risky drinking" or "substance misuse" or "substance use*" or "misuse substances" or "substance disorder" or "substance abuse" or "hazardous drinking" or "hazardous alcohol" or "harmful alcohol" or "harmful drinking" or "alcohol dependen*" or "dependent drinking" or alcoholic or alcoholism or "drug consumption" or "drug misuse" or "drug use*" or "misuse drugs" or "drug disorder*" or "drug dependen*" or "illicit drugs" or "alcohol or other drug*") adj2 (parent* or father* or paternal or step?father or mother* or maternal or step?mother or carer* or care?giver or foster?parent* or grand?parent* or grand?mother* or grand?father* or family or families)).mp.

"children of alcoholics"/

*Qualitative Research*

1 Qualitative Research.mp.

2 Interview.mp.

3 (theme$ or thematic).mp.

4 qualitative.af.

5 Nursing Methodology Research.mp.

6 questionnaire$.mp.

7 ethnological research.mp.

8 ethnograph$.mp.

9 ethnonursing.af.

10 phenomenol$.af.

11 (grounded adj (theor$ or study or studies or research or analys?s)).af.

12 (life stor$ or women* stor$).mp.

13 (emic or etic or hermeneutic$ or heuristic$ or semiotic$).af. or (data adj1 saturat$).tw. or participant observ$.tw.

14 (social construct$ or (postmodern$ or post-structural$) or (post structural$ or poststructural$) or post modern$ or post-modern$ or feminis$ or interpret$).mp.

15 (action research or cooperative inquir$ or co operative inquir$ or co-operative inquir$).mp.

16 (humanistic or existential or experiential or paradigm$).mp.

17 (field adj (study or studies or research)).tw.

18 human science.tw.

19 biographical method.tw.

20 theoretical sampl$.af.

21 ((purpos$ adj4 sampl$) or (focus adj group$)).af.

22 (account or accounts or unstructured or openended or open ended or text$ or narrative$).mp.

23 (life world or life-world or conversation analys?s or personal experience$ or theoretical saturation).mp.

24 ((lived or life) adj experience$).mp.

25 cluster sampl$.mp.

26 observational method$.af.

27 content analysis.af.

28 (constant adj (comparative or comparison)).af.

29 ((discourse$ or discurs$) adj3 analys?s).tw.

30 narrative analys?s.af.

31 heidegger$.tw.

32 colaizzi$.tw.

33 spiegelberg$.tw.

34 (van adj manen$).tw.

35 (van adj kaam$).tw.

36 (merleau adj ponty$).tw.

37 husserl$.tw.

38 foucault$.tw.

39 (corbin$ adj2 strauss$).tw.

40 glaser$.tw.

41 Living with.mp.

exp qualitative methods/ or exp "experiences (events)"/

Above combined with AND.

Found 6/6 papers in database.

**EBSCOhost (Cumulative Index to Nursing and Allied Health Literature)**

*Children and Young People*

"young person" or "young people" or young* or child* or youth or adolescen* or teen* or pre?teen or "young adult*" or offspring or juvenile* or pubescen* or "school child*" or student or boy* or girl* or pupil

(MH "Child+") OR (MH "Adolescence+") OR (MH "Young Adult")

*Parental Substance Use*

(("alcohol consumption" or "alcohol misuse" or "alcohol use*" or "misuse alcohol" or "alcohol intoxicat*" or "alcohol drinking" or "alcohol disorder*" or "binge drinking" or "social drinking" or "risky drinking" or "substance misuse" or "substance use*" or "misuse substances" or "substance disorder" or "substance abuse" or "hazardous drinking" or "hazardous alcohol" or "harmful alcohol" or "harmful drinking" or "alcohol dependen*" or "dependent drinking" or alcoholic or alcoholism or "drug consumption" or "drug misuse" or "drug use*" or "misuse drugs" or "drug disorder*" or "drug dependen*" or "illicit drugs" or "alcohol or other drug*") N2 (parent* or father* or paternal or step?father or mother* or maternal or step?mother or carer* or care?giver or foster?parent* or grand?parent* or grand?mother* or grand?father* or family or families))

(MH "Children of Alcoholics") OR (MH "Substance Abusers+")

*Qualitative Research*

1. Ethnonursing

2. ethnograph*

3. phenomenol*

4. grounded N1 theor*

5. grounded N1 study

6. grounded N1 studies

7. grounded N1 research

8. grounded N1 analys?s

9. life stor*

10. women’s stor*

11. emic or etic or hermeneutic$ or heuristic$ or semiotic$

12. data N1 saturat*

13. participant observ*

14. social construct* or postmodern* or post-structural* or post structural* or poststructural* or postmodern* or post-modern* or feminis* or interpret*

15. action research or cooperative inquir* or co operative inquir* or co-operative inquir*

16. humanistic or existential or experiential or paradigm*

17. field N1 stud*

18. field N1 research

19. human science

20. biographical method

21. theoretical sampl*

22. purpos* N4 sampl*

23. focus N1 group*

24. account or accounts or unstructured or openended or open ended or text* or narrative*

25. life world or life-world or conversation analys?s or personal experience* or theoretical saturation

26. lived experience*

27. life experience*

28. cluster sampl*

29. theme* or thematic

30. observational method*

31. questionnaire*

32. content analysis

33. discourse* N3 analys?s

34. discurs* N3 analys?s

35. constant N1 comparative

36. constant N1 comparison

37. narrative analys?s

38. Heidegger*

39. Colaizzi*

40. Spiegelberg*

41. van N1 manen*

42. van N1 kaam*

43. merleau N1 ponty*

44. husserl*

45. Foucault*

46. Corbin* N2 strauss*

47. glaser*

48. living with

(MH Interview+) OR (MH audiorecording) OR (MH Interviews+) OR (MH "Grounded theory") OR (MH "Qualitative Studies") OR (MH "Research, Nursing") OR (MH Questionnaires+) OR (MH "Focus Groups") OR (MH "Discourse Analysis") OR (MH "Content Analysis") OR (MH "Ethnographic Research") OR (MH "Ethnological Research") OR (MH "Ethnonursing Research") OR (MH "Constant Comparative Method") OR (MH "Qualitative Validity+") OR (MH "Purposive Sample") OR (MH "Observational Methods+") OR (MH "Field Studies") OR (MH "theoretical sample") OR (MH Phenomenology) OR (MH "Phenomenological Research") OR (MH "Life Experiences+") OR (MH "Cluster Sample+")

Above combined with AND.

Found 5/5 papers in database.

**Scopus**

*Children and Young People*

TITLE-ABS("young person" OR "young people" OR young* OR child* OR youth OR adolescen* OR teen* OR pre?teen OR "young adult*" OR offspring OR juvenile* OR pubescen* OR "school child*" OR student OR boy* OR girl* OR pupil)

*Parental Substance Use*

TITLE-ABS((("alcohol consumption" OR "alcohol misuse" OR "alcohol use*" OR "misuse alcohol" OR "alcohol intoxicat*" OR "alcohol drinking" OR "alcohol disorder*" OR "binge drinking" OR "social drinking" OR "risky drinking" OR "substance misuse" OR "substance use*" OR "misuse substances" OR "substance disorder" OR "substance abuse" OR "hazardous drinking" OR "hazardous alcohol" OR "harmful alcohol" OR "harmful drinking" OR "alcohol dependen*" OR "dependent drinking" OR alcoholic OR alcoholism OR "drug consumption" OR "drug misuse" OR "drug use*" OR "misuse drugs" OR "drug disorder*" OR "drug dependen*" OR "illicit drugs" OR "alcohol or other drug*") W/2 (parent* OR father* OR paternal OR step?father OR mother* OR maternal OR step?mother OR carer* OR care?giver OR foster?parent* OR grand?parent* OR grand?mother* OR grand?father* OR family OR families)))

*Qualitative Research*

TITLE-ABS(interview* OR theme* OR "thematic analysis" OR qualitative OR "nursing research methodology" OR questionnaire OR ethnograph* OR ethnonursing OR "ethnological research" OR phenomenol* OR "grounded theor*" OR "grounded stud*" OR "grounded research" OR "grounded analys?s" OR "Life Stor*" OR "Women's Stor*" OR emic OR etic OR hermeneutic OR heuristic OR semiotic OR "data saturat*" OR "participant observ*" OR "social construct*" OR Postmodern* OR "Post structural*" OR feminis* OR interpret* OR "action research" OR "co-operative inquir*" OR Humanistic OR Existential OR Experiential OR Paradigm* OR "field stud*" OR "field research" OR "human science" OR "biographical method" OR "theoretical sampl*" OR "Purposive sampl*" OR "open-ended account*" OR "unstructured account" OR narrative* OR text* OR "life world" OR "conversation analys?s" OR "theoretical saturation" OR "lived experience" OR "life experience" OR "living with" OR "cluster sampl*" OR "observational method*" OR "content analysis" OR "constant comparative" OR "discourse analys?s" OR "discurs* analys?s" OR "narrative analys?s" OR Heidegger* OR colaizzi* OR spiegelberg* OR "Van manen*" OR "Van Kaam*" OR "merleau ponty*" OR Husserl* OR Foucault* OR corbin* OR strauss* OR glaser*)

Above combined with AND.

Found 7/7 papers in database.

**Supplemental Appendix B. Quality appraisal of included studies using the 10-item Critical Appraisal Skills Programme (CASP) Qualitative Studies Checklist. Key: Yes (**🗸**), No (X), Cannot tell (?)**

| **First Author (year)** | **Aim** | **Method** | **Design** | **Recruitment** | **Data Collection** | **Researcher Bias** | **Ethics** | **Analysis** | **Findings** | **Value** |
| --- | --- | --- | --- | --- | --- | --- | --- | --- | --- | --- |
| Ahuja (2003) | 🗸 | 🗸 | ? | 🗸 | 🗸 | ? | ? | 🗸 | 🗸 | ? |
| Alexanderson (2016) | 🗸 | 🗸 | ? | 🗸 | 🗸 | ? | ? | 🗸 | 🗸 | ? |
| Bancroft (2004) | 🗸 | 🗸 | 🗸 | 🗸 | 🗸 | 🗸 | 🗸 | 🗸 | 🗸 | 🗸 |
| Barnard (2003) | 🗸 | 🗸 | ? | 🗸 | 🗸 | X | ? | ? | 🗸 | 🗸 |
| Bickelhaupt (2021) | 🗸 | 🗸 | ? | 🗸 | 🗸 | ? | ? | 🗸 | 🗸 | 🗸 |
| Christensen (1997) | 🗸 | 🗸 | ? | ? | ? | X | ? | ? | ? | 🗸 |
| D’Costa (2021) | 🗸 | 🗸 | 🗸 | 🗸 | 🗸 | 🗸 | ? | ? | 🗸 | 🗸 |
| Dundas (2000) | 🗸 | 🗸 | 🗸 | ? | 🗸 | ? | ? | X | 🗸 | ? |
| Fraser (2009) | 🗸 | 🗸 | 🗸 | 🗸 | 🗸 | X | 🗸 | ? | 🗸 | 🗸 |
| Hagstrom (2019) | 🗸 | 🗸 | 🗸 | ? | 🗸 | ? | 🗸 | 🗸 | 🗸 | 🗸 |
| Hill (1996) | 🗸 | 🗸 | ? | 🗸 | 🗸 | X | ? | X | ? | 🗸 |
| Hill (2015) | 🗸 | 🗸 | 🗸 | 🗸 | 🗸 | X | 🗸 | ? | 🗸 | 🗸 |
| Holmila (2011) | 🗸 | 🗸 | 🗸 | ? | 🗸 | X | 🗸 | ? | 🗸 | 🗸 |
| Houmøller (2011) | 🗸 | 🗸 | 🗸 | 🗸 | 🗸 | X | 🗸 | ? | 🗸 | 🗸 |
| Johnson (2013) | 🗸 | 🗸 | 🗸 | 🗸 | 🗸 | X | 🗸 | 🗸 | 🗸 | 🗸 |
| McGuire (2002) | 🗸 | 🗸 | 🗸 | 🗸 | ? | X | 🗸 | ? | 🗸 | 🗸 |
| McLaughlin (2015) | 🗸 | 🗸 | 🗸 | 🗸 | 🗸 | X | 🗸 | X | ? | 🗸 |
| Moore (2010) | 🗸 | 🗸 | 🗸 | 🗸 | 🗸 | X | 🗸 | 🗸 | 🗸 | 🗸 |
| Mudau (2018) | 🗸 | 🗸 | 🗸 | ? | ? | X | 🗸 | ? | ? | 🗸 |
| Murray (1998) | 🗸 | 🗸 | 🗸 | ? | 🗸 | 🗸 | 🗸 | 🗸 | 🗸 | 🗸 |
| Nattala (2020) | 🗸 | 🗸 | 🗸 | 🗸 | 🗸 | X | 🗸 | ? | 🗸 | 🗸 |
| O’Connor (2014) | 🗸 | 🗸 | 🗸 | 🗸 | 🗸 | X | 🗸 | ? | 🗸 | 🗸 |
| Offiong (2020) | 🗸 | 🗸 | 🗸 | 🗸 | 🗸 | 🗸 | 🗸 | 🗸 | 🗸 | 🗸 |
| Park (2016) | 🗸 | 🗸 | 🗸 | 🗸 | 🗸 | ? | ? | 🗸 | 🗸 | 🗸 |
| Ramirez (2014) | 🗸 | 🗸 | 🗸 | ? | ? | X | ? | X | 🗸 | 🗸 |
| Reupert (2012) | 🗸 | 🗸 | 🗸 | 🗸 | 🗸 | 🗸 | 🗸 | 🗸 | 🗸 | 🗸 |
| Ronel (2010) | 🗸 | 🗸 | 🗸 | 🗸 | 🗸 | X | 🗸 | 🗸 | 🗸 | 🗸 |
| Silva (2013a) | 🗸 | 🗸 | 🗸 | ? | 🗸 | ? | 🗸 | 🗸 | 🗸 | ? |
| Tamutiené (2019) | 🗸 | 🗸 | ? | 🗸 | 🗸 | X | 🗸 | 🗸 | 🗸 | 🗸 |
| Tinnält (2011) | 🗸 | 🗸 | ? | ? | ? | X | 🗸 | 🗸 | 🗸 | 🗸 |
| Tinnfält (2018) | 🗸 | 🗸 | 🗸 | 🗸 | 🗸 | ? | 🗸 | 🗸 | 🗸 | 🗸 |
| Turning Point (2006) | 🗸 | 🗸 | ? | ? | ? | X | ? | ? | 🗸 | 🗸 |
| Velleman (2008) | 🗸 | 🗸 | 🗸 | 🗸 | 🗸 | X | 🗸 | ? | X | ? |
| Wangensteen (2019a) | 🗸 | 🗸 | 🗸 | ? | 🗸 | X | 🗸 | 🗸 | 🗸 | 🗸 |
| Yusay (2019) | 🗸 | 🗸 | 🗸 | 🗸 | ? | 🗸 | 🗸 | 🗸 | 🗸 | 🗸 |

**Supplemental Appendix C. Table showing which studies are related to each theme and sub-theme.**

| **First Author (Year)** | **Theme 1: Unpredictability** | | | | **Theme 2: Impacts** | | | | **Theme 3: Control/agency** | | **Theme 4: Coping/resisting** | | **Theme 5: Support** | | |
| --- | --- | --- | --- | --- | --- | --- | --- | --- | --- | --- | --- | --- | --- | --- | --- |
|  | **1** | **2** | **3** | **4** | **1** | **2** | **3** | **4** | **1** | **2** | **1** | **2** | **1** | **2** | **3** |
| Ahuja (2003) | 🗸 | 🗸 | 🗸 | 🗸 | 🗸 | 🗸 | 🗸 |  | 🗸 | 🗸 | 🗸 |  |  | 🗸 |  |
| Alexanderson (2016) | 🗸 | 🗸 | 🗸 | 🗸 | 🗸 | 🗸 |  |  | 🗸 | 🗸 | 🗸 | 🗸 | 🗸 |  | 🗸 |
| Bancroft (2004)* | 🗸 | 🗸 | 🗸 | 🗸 | 🗸 | 🗸 | 🗸 | 🗸 | 🗸 | 🗸 | 🗸 | 🗸 | 🗸 | 🗸 | 🗸 |
| **Backett-Milburn, 2008* | 🗸 | 🗸 | 🗸 | 🗸 | 🗸 | 🗸 | 🗸 | 🗸 | 🗸 | 🗸 | 🗸 | 🗸 | 🗸 | 🗸 |  |
| **Wilson, 2008* |  | 🗸 |  | 🗸 | 🗸 | 🗸 |  |  |  |  | 🗸 | 🗸 | 🗸 | 🗸 |  |
| **Wilson, 2012* | 🗸 |  |  | 🗸 |  | 🗸 | 🗸 |  | 🗸 |  |  | 🗸 | 🗸 |  |  |
| Barnard (2003) | 🗸 |  |  |  |  | 🗸 | 🗸 | 🗸 | 🗸 | 🗸 | 🗸 | 🗸 |  | 🗸 |  |
| Bickelhaupt (2021) | 🗸 | 🗸 |  |  | 🗸 | 🗸 | 🗸 |  | 🗸 |  | 🗸 |  |  | 🗸 | 🗸 |
| Christensen (1997) | 🗸 | 🗸 |  |  |  | 🗸 | 🗸 |  | 🗸 | 🗸 |  | 🗸 |  |  | 🗸 |
| D’Costa (2021) | 🗸 | 🗸 | 🗸 |  | 🗸 | 🗸 | 🗸 |  | 🗸 | 🗸 | 🗸 | 🗸 | 🗸 | 🗸 | 🗸 |
| Dundas (2000) |  |  |  | 🗸 | 🗸 | 🗸 | 🗸 |  | 🗸 | 🗸 | 🗸 |  | 🗸 | 🗸 |  |
| Fraser (2009) | 🗸 | 🗸 | 🗸 | 🗸 | 🗸 | 🗸 | 🗸 | 🗸 | 🗸 | 🗸 | 🗸 |  | 🗸 |  | 🗸 |
| Hagström (2019) |  | 🗸 | 🗸 | 🗸 | 🗸 | 🗸 | 🗸 | 🗸 | 🗸 | 🗸 | 🗸 | 🗸 | 🗸 | 🗸 |  |
| Hill (1996) | 🗸 | 🗸 | 🗸 |  | 🗸 | 🗸 | 🗸 | 🗸 | 🗸 | 🗸 |  | 🗸 | 🗸 | 🗸 | 🗸 |
| Hill (2015) | 🗸 | 🗸 |  | 🗸 | 🗸 | 🗸 |  |  | 🗸 |  | 🗸 | 🗸 | 🗸 | 🗸 | 🗸 |
| Holmila (2011) |  |  | 🗸 |  | 🗸 | 🗸 | 🗸 | 🗸 | 🗸 | 🗸 | 🗸 | 🗸 | 🗸 | 🗸 | 🗸 |
| Houmøller (2011)* | 🗸 | 🗸 | 🗸 | 🗸 | 🗸 | 🗸 | 🗸 | 🗸 | 🗸 | 🗸 |  | 🗸 | 🗸 | 🗸 | 🗸 |
| **Bernays, 2011* | 🗸 | 🗸 | 🗸 | 🗸 |  | 🗸 | 🗸 |  | 🗸 | 🗸 |  | 🗸 | 🗸 |  | 🗸 |
| Johnson (2013) | 🗸 | 🗸 | 🗸 | 🗸 | 🗸 | 🗸 |  |  | 🗸 | 🗸 |  | 🗸 | 🗸 |  | 🗸 |
| McGuire (2002) | 🗸 | 🗸 | 🗸 | 🗸 | 🗸 | 🗸 | 🗸 | 🗸 | 🗸 | 🗸 |  | 🗸 | 🗸 |  | 🗸 |
| McLaughlin (2015) |  |  | 🗸 | 🗸 |  | 🗸 |  |  |  | 🗸 |  | 🗸 | 🗸 | 🗸 | 🗸 |
| Moore (2010)* | 🗸 | 🗸 | 🗸 | 🗸 | 🗸 | 🗸 | 🗸 | 🗸 | 🗸 | 🗸 | 🗸 | 🗸 | 🗸 | 🗸 | 🗸 |
| **Moore, 2011* | 🗸 | 🗸 | 🗸 | 🗸 |  | 🗸 |  | 🗸 | 🗸 | 🗸 |  |  |  | 🗸 | 🗸 |
| Mudau (2018) |  | 🗸 |  |  | 🗸 | 🗸 | 🗸 | 🗸 |  | 🗸 |  | 🗸 | 🗸 | 🗸 | 🗸 |
| Murray (1998) | 🗸 | 🗸 | 🗸 | 🗸 |  | 🗸 | 🗸 |  |  |  | 🗸 | 🗸 |  |  | 🗸 |
| Nattala (2020) | 🗸 | 🗸 | 🗸 | 🗸 | 🗸 | 🗸 | 🗸 | 🗸 | 🗸 | 🗸 | 🗸 | 🗸 | 🗸 | 🗸 | 🗸 |
| O’Connor (2014) | 🗸 | 🗸 | 🗸 | 🗸 | 🗸 | 🗸 | 🗸 |  | 🗸 |  | 🗸 |  | 🗸 | 🗸 | 🗸 |
| Offiong (2020)* |  | 🗸 | 🗸 | 🗸 | 🗸 | 🗸 | 🗸 | 🗸 |  |  | 🗸 |  | 🗸 |  |  |
| **Lewis, 2021* |  |  | 🗸 | 🗸 | 🗸 | 🗸 |  | 🗸 |  |  | 🗸 |  | 🗸 | 🗸 | 🗸 |
| **Powell, 2021* |  |  |  |  | 🗸 |  |  |  |  | 🗸 |  | 🗸 | 🗸 |  | 🗸 |
| Park (2016)* | 🗸 | 🗸 | 🗸 | 🗸 | 🗸 | 🗸 | 🗸 | 🗸 | 🗸 | 🗸 | 🗸 | 🗸 |  |  |  |
| **Park, 2017* | 🗸 | 🗸 |  |  | 🗸 | 🗸 | 🗸 |  | 🗸 | 🗸 |  |  |  | 🗸 | 🗸 |
| **Park 2018* |  |  |  | 🗸 | 🗸 |  |  | 🗸 |  | 🗸 |  | 🗸 |  | 🗸 | 🗸 |
| Ramirez (2014) | 🗸 | 🗸 | 🗸 |  | 🗸 | 🗸 |  | 🗸 | 🗸 | 🗸 |  |  |  | 🗸 |  |
| Reupert (2012) | 🗸 |  | 🗸 | 🗸 | 🗸 | 🗸 | 🗸 | 🗸 | 🗸 |  |  | 🗸 |  |  | 🗸 |
| Ronel (2010)* |  |  | 🗸 | 🗸 | 🗸 | 🗸 | 🗸 |  | 🗸 | 🗸 | 🗸 |  | 🗸 | 🗸 |  |
| **Ronel, 2011* |  |  | 🗸 |  |  | 🗸 |  | 🗸 |  |  | 🗸 |  | 🗸 | 🗸 |  |
| Silva (2013a)* |  | 🗸 |  |  | 🗸 | 🗸 |  | 🗸 |  | 🗸 |  |  |  |  |  |
| **Silva, 2013b* | 🗸 | 🗸 |  |  |  |  |  | 🗸 |  |  |  |  |  |  |  |
| Tamutiené (2019) |  |  |  | 🗸 | 🗸 | 🗸 | 🗸 | 🗸 |  | 🗸 | 🗸 | 🗸 | 🗸 | 🗸 | 🗸 |
| Tinnfält (2011) |  |  |  |  |  | 🗸 |  |  |  |  | 🗸 | 🗸 | 🗸 | 🗸 | 🗸 |
| Tinnfält (2018) | 🗸 | 🗸 |  | 🗸 | 🗸 | 🗸 | 🗸 |  | 🗸 | 🗸 | 🗸 | 🗸 |  |  | 🗸 |
| Turning Point (2006) | 🗸 | 🗸 | 🗸 | 🗸 | 🗸 | 🗸 | 🗸 | 🗸 | 🗸 | 🗸 | 🗸 | 🗸 | 🗸 | 🗸 | 🗸 |
| Velleman* (2008) |  | 🗸 |  |  | 🗸 | 🗸 |  |  | 🗸 | 🗸 | 🗸 | 🗸 |  |  | 🗸 |
| **Templeton, 2009* |  | 🗸 | 🗸 | 🗸 | 🗸 | 🗸 |  | 🗸 | 🗸 | 🗸 | 🗸 | 🗸 |  | 🗸 | 🗸 |
| Wangensteen (2019a)* | 🗸 | 🗸 |  |  | 🗸 | 🗸 | 🗸 |  | 🗸 |  |  | 🗸 |  |  | 🗸 |
| **Wangensteen, 2019b* | 🗸 | 🗸 |  | 🗸 | 🗸 | 🗸 | 🗸 |  | 🗸 |  |  |  | 🗸 | 🗸 |  |
| **Wangensteen, 2020* |  | 🗸 |  |  | 🗸 |  | 🗸 |  |  |  |  | 🗸 |  |  | 🗸 |
| Yusay (2019) |  | 🗸 |  |  | 🗸 | 🗸 | 🗸 | 🗸 | 🗸 | 🗸 |  | 🗸 |  |  |  |
